# Overactive Neuronal eEF2K/eEF2 signaling is associated with cognitive impairment and apathy-like behavior

**DOI:** 10.1038/s41380-025-03408-z

**Published:** 2025-12-14

**Authors:** Hannah M. Jester, Noelle Nicol, Qian Yang, Yankai Zhang, Amelia Suhocki, Xueyan Zhou, Ethan Underwood, Hadi Pourhadi, Dongmei Cheng, Jingyun Lee, Peiqing Sun, Cristina M. Furdui, Christopher G. Proud, Kobi Rosenblum, Tao Ma

**Affiliations:** 1 Department of Internal Medicine-Gerontology and Geriatric Medicine, Wake Forest School of Medicine, Winston-Salem, NC 27157 USA; 2 Department of Internal Medicine-Section on Molecular Medicine, Wake Forest School of Medicine, Winston-Salem, NC 27157 USA; 3 Department of Cancer Biology, Wake Forest Baptist Comprehensive Cancer Center, Wake Forest School of Medicine, Winston-Salem, NC 27157 USA; 4 Lifelong Health, South Australian Health and Medical Research Institute, Adelaide, SA 5001 Australia; 5 Sagol Department of Neurobiology, University of Haifa, 3498838 Haifa, Israel; 6 Department of Translational Neuroscience, Wake Forest School of Medicine, Winston-Salem, NC 27157 USA

**Keywords:** Neuroscience, Diseases

## Abstract

Dysregulated protein synthesis has been implicated in multiple neurodevelopmental, neurodegenerative, and neuropsychiatric diseases. Protein synthesis or mRNA translation is critically regulated through phosphorylation of eukaryotic elongation factor 2 (eEF2) by its kinase eEF2K. Increased eEF2K activity leads to elevated phosphorylation and inhibition of eEF2 and inhibits the elongation phase of protein synthesis. Recent studies suggest a link between eEF2 hyper-phosphorylation and several neuronal diseases characterized by cognitive impairments. Phosphorylation of eEF2 by eEF2K has also been implicated as a molecular mechanism for the rapid antidepressant effect of ketamine. Whether there exists a causal relationship between overactive eEF2K/eEF2 signaling and impaired synaptic and cognitive function remains unknown. To fill this critical knowledge gap, we generated a transgenic mouse model (eEF2K-cKI) overexpressing eEF2K in excitatory neurons to investigate how eEF2K/eEF2 signaling can impact cognitive functions and neuropsychiatric behaviors. We assessed hippocampal-dependent learning and memory, as well as multiple neuropsychiatric domains associated with a depressive phenotype including despair, anhedonia, apathy, anxiety, and sociability. The eEF2K-cKI mice exhibit learning and memory impairments, and robust apathy-like phenotype without other despair/depression-like behaviors. We also found impaired long-term potentiation and altered dendritic spine and synaptic morphology in the hippocampus of the eEF2K-cKI mice. Proteomic analysis revealed changes in levels of proteins associated with neuropsychiatric and neurodegenerative disorders. Our findings present direct evidence supporting the pathophysiological role of aberrant eEF2K/eEF2 signaling in brain function and help provide insight into novel mechanisms and therapeutic avenues for neuronal diseases characterized by dementia and neuropsychiatric symptoms.

## Introduction

Dysregulated mRNA translation (protein synthesis) has been implicated in multiple neurodevelopmental, neurodegenerative, and neuropsychiatric diseases such as Down Syndrome (DS), Alzheimer’s disease (AD), and depression [1–6]. Protein synthesis is a highly regulated process that involves numerous signaling pathways and translation factors particularly regulating the initiation and elongation stages [7, 8]. This regulation allows for complex changes to the proteome and increases in specific mRNAs in response to different cellular environments [9–12]. While the initiation phase is considered as the “rate-limiting step” and draws the most attention in studying translational control, accumulating evidence indicates that regulation of elongation plays a critical role in the protein synthesis process during cellular responses to deficiency of energy and nutrients [13]. Furthermore, the energy and amino acids used in protein synthesis are mostly ( > 95%) consumed during the elongation phase [14, 15]. One critical mechanism for elongation regulation is through the eukaryotic elongation factor 2 kinase (eEF2K). eEF2K is a Ca^2+^/calmodulin-dependent α-kinase and acts specifically to phosphorylate its only known substrate eukaryotic elongation factor 2 (eEF2) at T56 and inhibit its activity and thus protein synthesis [15]. eEF2 is a GTPase that catalyzes the translocation of the tRNA from the A site to the P site in the ribosome facilitating the growing peptide chain and regulates the elongation step of protein synthesis [15]. Activated eEF2K increases eEF2 phosphorylation and inhibits the elongation step of protein synthesis [15]. This process is associated with the nutrient and energy state of the cell via its regulation by the mammalian target of rapamycin complex 1 (mTORC1) and AMP-activated protein kinase (AMPK) [16]. mTORC1 is a nutrient sensor and is inhibited when nutrients like amino acids, the building blocks of proteins, are low [17]. mTORC1 signaling is responsible for phosphorylation of multiple inhibitory sites on eEF2K either directly by mTORC1 or by its downstream substrates such as ribosomal protein S6 kinase 1 [15, 16]. AMPK is an energy sensory that is activated by low energy levels [18, 19]. It interacts with the mTORC1 pathway at multiple points to affect protein synthesis. AMPK can activate TSC2, an upstream inhibitor of mTORC1 [20]. AMPK inhibits Raptor, an mTORC1 constituent, leading to inactivation of mTORC1 signaling [21]. AMPK can also directly phosphorylate and activate eEF2K [22–24]. Overall, eEF2K is activated under conditions of cellular stress [11, 25–28].

Regulation of protein synthesis may be a crucial aspect of major depressive disorder (MDD) and other mood disorders. A recent study found that anisomycin, a general protein synthesis inhibitor, induced depressive-like behaviors in mice [29]. Additionally, increased eIF2α phosphorylation and decreased eIF2B, i.e. inhibition of translation initiation, was also linked to depressive-like behaviors in humans and mice [29]. Another recent study reported altered tRNA dynamics in suicide (MDD associated) brain [30]. Notably, recent studies have implicated a role of eEF2K signaling regulation in mediating the antidepressant effects of ketamine [6, 31]. Ketamine may exert its antidepression effects by inhibiting eEF2K and eEF2 phosphorylation, leading to an increase in translation [6, 31]. Genetic deletion of eEF2K abolishes ketamine’s antidepressant effect [31]. In agreement with these findings, eEF2K overactivation and eEF2 hyper-phosphorylation, as well as decreased protein synthesis, have been linked to MDD [1, 32]. Therefore, we hypothesize that increased eEF2K expression and eEF2 phosphorylation could promote a depression/despair-like phenotype as well as learning and memory deficits.

Here, we generated a novel transgenic mouse model with neuron-specific eEF2K overexpression to investigate whether and how overactive eEF2K/eEF2 signaling in the mature brain can impact psychological and cognitive functions. We assessed multiple neuropsychiatric domains associated with a depression-like phenotype including despair, anhedonia, apathy, anxiety, and sociability. We also assessed hippocampal-dependent learning and memory and evaluated the role of eEF2K in synaptic plasticity and synaptic function and structure. In addition, we performed a proteomic analysis to identify proteins dysregulated by eEF2K overexpression to better understand how eEF2K signaling might contribute to the synaptic and behavioral changes.

## Results

### Generation of the eEF2K overexpression mouse model

To understand the role of eEF2K and eEF2 phosphorylation in mammalian neurons in regulating synaptic and cognitive function, we generated a conditional eEF2K knock-in model (referred as eEF2K-cKI hereafter). Briefly, we first created a line of transgenic mouse with *Eef2K* knock-in flanked by a *lox*STOP*lox* cassette using the CRISPR/Cas9-mediated genome editing combined with a *Rosa26* knock-in strategy [33] (Supplemental Fig. 1A). We then crossed these mice with the transgenic mice expressing a neuron-specific Cre recombinase (Camk2a-cre) [34] to remove the STOP cassette and thus induce conditional overexpression of eEF2K in excitatory neurons in the forebrain and hippocampus (Supplemental Fig. 1B). Overexpression of eEF2K was confirmed by both the mRNA and protein levels measured by RT-PCR and western blot respectively (Supplemental Figs. 1C and D). As expected, an increased eEF2K expression resulted in a significant increase in eEF2 phosphorylation at the T56 site (Supplemental Fig. 1E). Both male and female mice, 3-5 months old, were used for all experiments.

### eEF2K overexpression does not lead to despair-like behavior but instead elicits a robust apathy-like phenotype

**Fig. 1:**
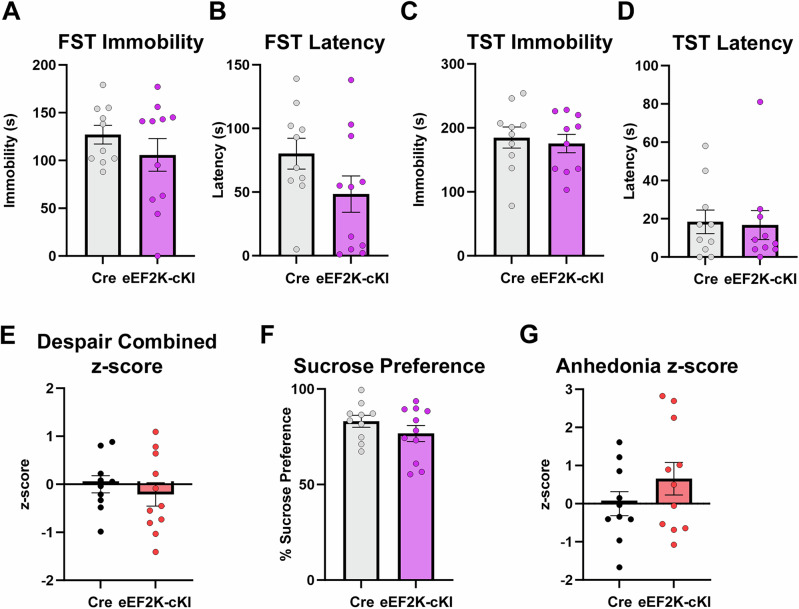
eEF2K overexpression did not affect despair or anhedonia-like behavior. (**A**) Total immobility time in FST was not different between groups. Cre, n = 10; eEF2K-cKI, n = 11. p = 0.3079. (**B**) Latency to immobility in FST was not significantly different between groups. Cre, n = 10; eEF2K-cKI, n = 11. p = 0.1094. (**C**) Total immobility time in TST was not different between groups. Cre, n = 10; eEF2K-cKI, n = 10. p = 0.6726. (**D**) Latency to immobility in TST was not significantly different between groups. Cre, n = 10; eEF2K-cKI, n = 10. p = 0.8638. (**E**) No significant difference between groups in the combined despair z-score. Cre, n = 10; eEF2K-cKI, n = 11. p = 0.4985. (**F**) Percent sucrose preference on day 3 was not different between groups. Cre, n = 10; eEF2K-cKI, n = 11. p = 0.2413. (**G**) There was no significant difference in the anhedonia z-score. Cre, n = 10; eEF2K-cKI, n = 11. p = 0.2413. Error bars represent ± SEM. Student’s T-test.

Abnormal translational control is linked to neuropsychiatric disorders and neurodegenerative diseases that are characterized or accompanied by multiple neuropsychiatric symptoms (NPS) [16, 35–37]. In preclinical models, depression-like phenotypes are often determined by assessing despair-like behavior (DLB) and anhedonia [38]. We first evaluated the DLB in the eEF2K-cKI mice using the forced swim test (FST) and tail suspension test (TST) [31]. FST immobility is the time spent immobile during the last 4 minutes of the trial, while latency is the first instance of immobility after being placed in the water. In contrast to our hypothesis, there was no difference between the groups in either FST immobility or latency (Fig. 1A, B). The number and duration of bouts was also recorded where each bout is defined as the conversion between mobility and immobility [39]. No differences were noted between groups for FST bouts and the longest bout (Supplemental Figs. 2A and B). Consistent with the FST results, analysis of the TST revealed no differences between the two groups in immobility (Fig. 1C), latency (Fig. 1D), and duration of the longest bout (Supplemental Fig. 2D). However, the eEF2K-cKI mice did exhibit more bouts in TST than the Cre controls (Supplemental Fig. 2C). Z-scores were calculated separately for each behavioral assay for each mouse [37]. A composite z-score was calculated as the average z-score for each mouse from multiple tests for each behavioral domain (i.e. despair-like behavior, apathy-like behavior, anxiety-like behavior). The composite score was used for assessment of overall despair-like behavior and showed no difference between the eEF2K-cKI mice and Cre controls (Fig. 1E). We assessed anhedonia using the sucrose preference test [40]. There was no difference in sucrose preference between eEF2K-cKI mice and Cre controls (Fig. 1F) and both groups had similar total consumption amounts (Supplemental Fig. 2E). Analysis of the z-score demonstrates no anhedonia-like behavior in the eEF2K-cKI mice (Fig. 1G). Therefore, in contrast to our hypothesis, these findings suggest that overactive eEF2K signaling and eEF2 hyper-phosphorylation are not associated with DLB or anhedonia.

**Fig. 2:**
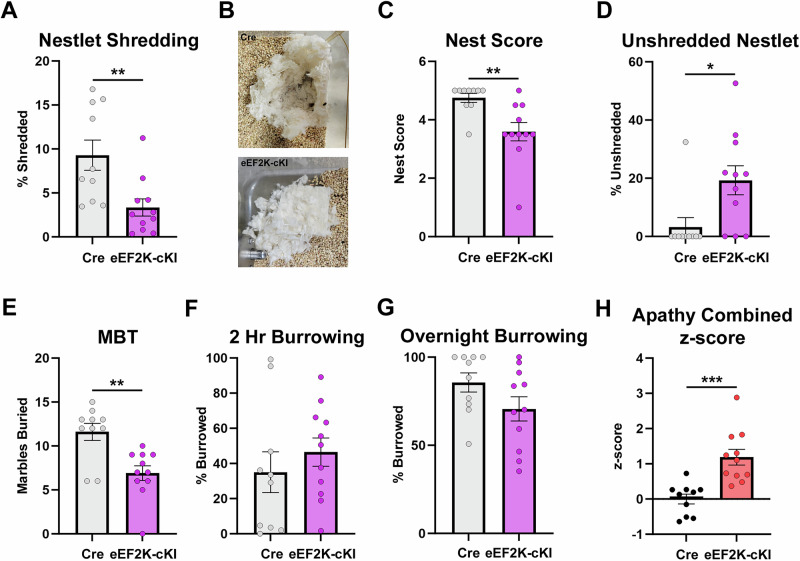
eEF2K overexpression increased apathy-like behavior. (**A**) The percent of nestlet shredded in a 30 min period was decreased in the eEF2K-cKI mice. Cre, n = 10; eEF2K-cKI, n = 11. p = 0.0060. (**B**) Representative images of nests for nest score (**C**) Nest building score was decreased in the eEF2K-cKI mice. Cre, n = 10; eEF2K-cKI, n = 11. p = 0.0047. (**D**) The percent of nestlet left unshredded during the nest building task was increased in the eEF2K-cKI mice. Cre, n = 10; eEF2K-cKI, n = 11. p = 0.0160. (**E**) The number of marbles buried in a 30 min period was decreased in the eEF2K-cKI mice. Cre, n = 10; eEF2K-cKI, n = 11. p = 0.0017. (**F**) There was no difference in the percent burrowed in the 2 hr burrowing task. Cre, n = 10; eEF2K-cKI, n = 11. p = 0.4232. (**G**) There was no difference in the percent burrowed in the overnight burrowing task. Cre, n = 10; eEF2K-cKI, n = 11. p = 0.1077. (**H**) The eEF2K-cKI mice had significantly higher combined apathy z-score compared to cre controls. Cre, n = 10; eEF2K-cKI, n = 11. p = 0.0003. Error bars represent ± SEM. *p < 0.05, **p < 0.01, ***p < 0.001; Student’s T-test.

Anhedonia and DLB are often co-occurring with apathy, such as the case in neurodegenerative diseases [41]. We assessed apathy-like behavior (ALB) using rodent-typical behaviors as previously described [37, 42]. Briefly, mice were subjected to 5 behavioral tasks including nestlet shredding (NS), nest building (NB), marble burying (MB), and burrowing (2 h and overnight). First, we looked at nestlet shredding and nest building behavior. There was a significant decrease in the amount of nestlet shredded in a 30-minute period in the eEF2K-cKI mice (Fig. 2A). Similarly, in the nest building task, the eEF2K-cKI mice made poorer nests with a lower nest score [37, 43], and left more of the nestlet unshredded after an overnight period (Fig. 2B-2D). In the marble burying task, the eEF2K-cKI mice buried fewer marbles than Cre controls (Fig. 2E). We did not observe significant differences between groups in 2 hour and overnight burrowing behavior tests (Figs. 2F and 2G). Finally, analysis of the composite z score revealed that the eEF2K-cKI mice showed a significantly higher apathy composite z-score indicating apathy-like behavior (Fig. 2H). These findings indicate that overactive eEF2K signaling results in apathy-like behavior.

**Fig. 3:**
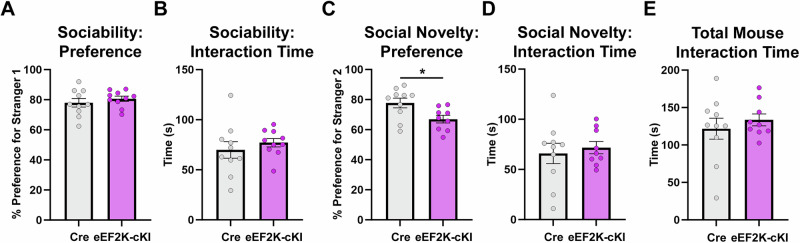
eEF2K overexpression decreased preference for social novelty in the 3-chamber sociability test. (**A**) No change in preference for stranger 1 over an object in the sociability portion of the task. Cre, n = 10; eEF2K-cKI, n = 10. p = 0.4248. (**B**) No change in total interaction time for both stranger 1 and object in the sociability portion of the task. Cre, n = 10; eEF2K-cKI, n = 10. p = 0.4453. (**C**) Decrease in stranger 2 preference over stranger 1 in eEF2K-cKI mice in the social novelty portion of the task. Cre, n = 10; eEF2K-cKI, n = 9. p = 0.0195. (**D**) No change in total interaction time for both stranger 1 and stranger 2 in the social novelty portion of the task. Cre, n = 10; eEF2K-cKI, n = 9. p = 0.6400. (**E**) No change in the total mouse interaction time (excluding time interacting with object) for both the sociability and social novelty portions of the task. Cre, n = 10; eEF2K-cKI, n = 9. p = 0.4895. Error bars represent ± SEM. *p < 0.05; Student’s T-test.

Changes in social behavior can also be associated with ALB, as well as a depressive phenotype [38, 44]. We evaluated the preference for social interaction and social novelty using the 3-chamber sociability task (3-CST) [45]. The eEF2K-cKI mice did not show any significant differences in the preference for mouse (stranger 1) over an object or in the total interaction time for the sociability portion of the test (Fig. 3A, B). However, when presented with a novel stranger mouse (stranger 2), eEF2K-cKI mice had a lower preference for Stranger 2 than Cre controls (Fig. 3C). The total interaction time for the social novelty portion of the test was not significantly different between groups and both groups spent similar amount of time interacting with a stranger mouse throughout both portions of the test (Figs. 3D and 3E). Such findings suggest that overactive eEF2K signaling and eEF2 phosphorylation leads to a decrease for social novelty preference but not in general sociability.

**Fig. 4:**
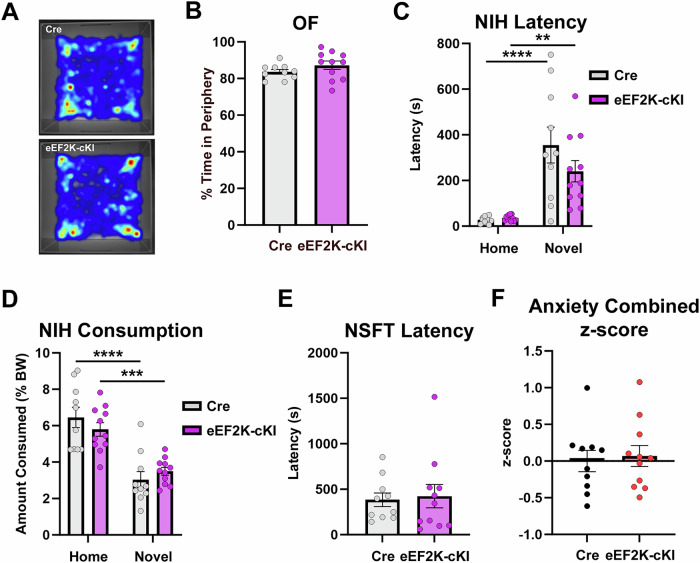
eEF2K overexpression had no effect on anxiety behaviors. (**A**) Representative heatmaps for OF. (**B**) Percent time spent in the periphery during OF was not significantly different between groups. Cre, n = 10; eEF2K-cKI, n = 11. Student’s T-test. p = 0.1955. (**C**) Latency to drink in the home cage (day 4) and in the novel cage (day 5) in the NIH task. There was no difference between groups in the novel cage. Cre, n = 10; eEF2K-cKI, n = 11. 2-way ANOVA. Cre Home vs Cre Novel p < 0.0001; eEF2K-cKI Home vs eEF2K-cKI Novel p = 0.0037; Cre Home vs eEF2K-cKI Home p = 0.8920; Cre Novel vs eEF2K-cKI Novel p = 0.0790. (**D**) Amount consumed as percent body weight in the home cage and novel cage in the NIH task. Both groups had decreased consumption in the novel cage but did not significantly differ from each other. Cre, n = 10; eEF2K-cKI, n = 11. 2-way ANOVA. Cre Home vs Cre Novel p < 0.0001; eEF2K-cKI Home vs eEF2K-cKI Novel p = 0.0001; Cre Home vs eEF2K-cKI Home p = 0.2563; Cre Novel vs eEF2K-cKI Novel p = 0.4342. (**E**) No difference in the latency to consume the food pellet in the NSFT. Cre, n = 10; eEF2K-cKI, n = 11. Student’s T-test. p = 0.7989. (**F**) There was no difference in the combined anxiety z-score between groups. Cre, n = 10; eEF2K-cKI, n = 11. Student’s T-test. p = 0.7376. Error bars represent ± SEM. **p < 0.01, ***p < 0.001, ****p < 0.0001; Student’s T-test and 2-way ANOVA.

Moreover, as a series of control experiments, we investigated anxiety-like behavior, which may be sensitive to eEF2K signaling and protein synthesis dysregulation [46, 47]. Anxiety-like behaviors may also confound ALB. In the open field (OF) task there was no significant difference between the eEF2K-cKI mice and the Cre controls for the percent time spent in the periphery (Fig. 4A, B). We performed two additional behavioral tests for evaluation of anxiety-like behavior including the novelty-induced hypophagia (NIH) and novelty suppressed feeding task (NSFT) [40, 48] For NIH, there was no difference in the latency to consume food or the amount consumed between groups in the home cage or in the novel environment, though both groups demonstrated the expected increase in anxiety in the novel environment (Figs. 4C and 4D; Supplemental Figs. 3A and 3B). There was also no difference in the latency to consume in the NSFT between groups (Fig. 4E). In addition, there was no difference in the amount consumed in the NSFT or in the weight change before and after food deprivation (Supplemental Figs. 3C and 3D). Finally, analysis of the composite anxiety z-score demonstrated no difference in anxiety-like behavior between the Cre and eEF2K-cKI mice (Fig. 4F). These data suggest that neuronal activation of eEF2K/eEF2 signaling does not result in anxiety-like behavior.

### eEF2K-cKI mice exhibit learning and memory impairments

**Fig. 5:**
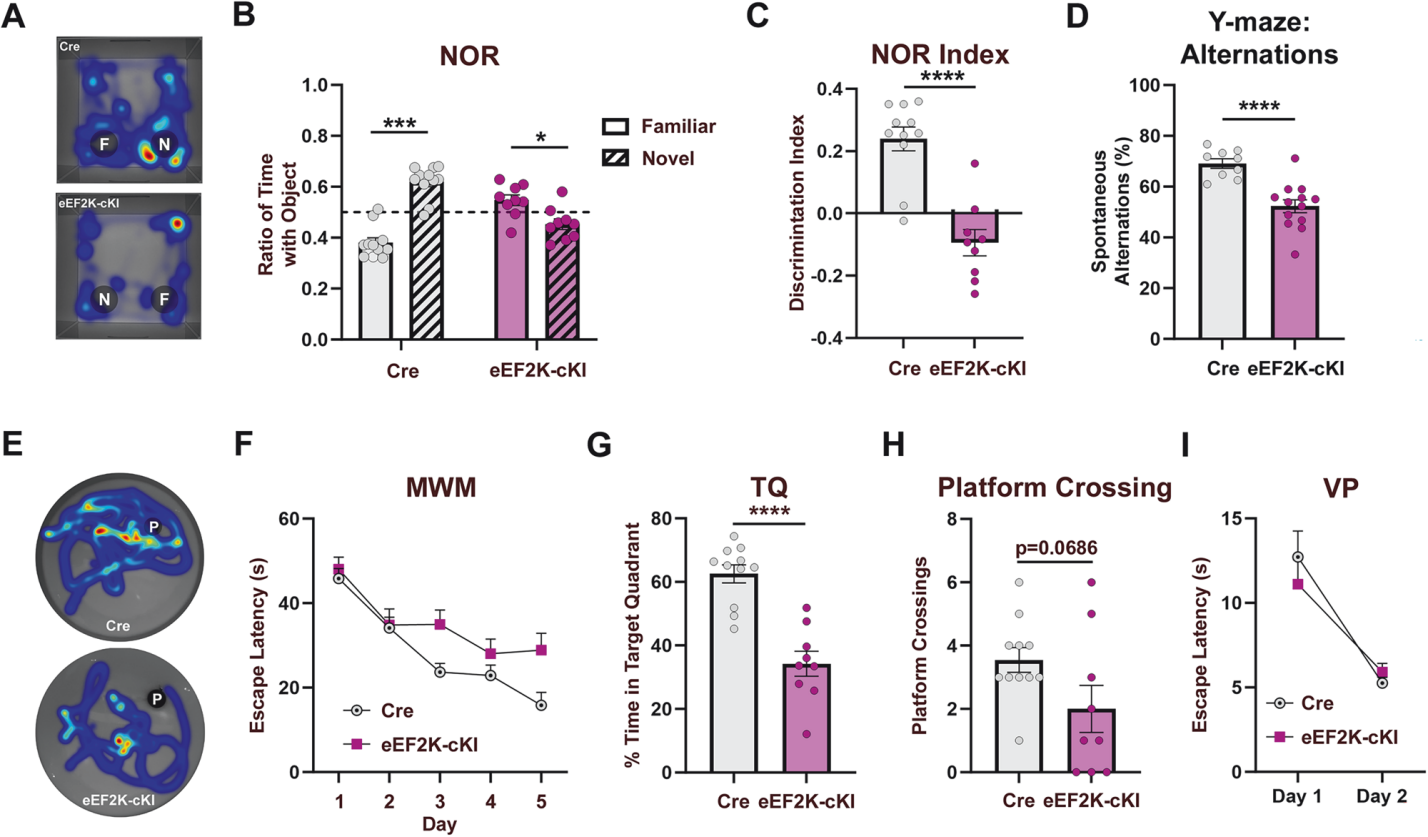
eEF2K overexpression impairs learning and memory. (**A**) Representative heatmaps during the NOR testing phase. “F” indicates familiar object. “N” indicates novel object. (**B**) Ratio of time spent with familiar (empty) and novel (stripped) objects in the NOR task during the testing phase. Preference of less than 0.5 for novel object indicates cognitive impairment as seen in the eEF2K-cKI mice. Cre Familiar vs Cre Novel p < 0.0001; eEF2K-cKI Familiar vs eEF2K-cKI Novel p = 0.0390 (**C**) Discrimination index, calculated as (time interacting with novel object – time interacting with familiar object) / total interaction time, was significantly decreased in eEF2K-cKI mice. Cre, n = 11; eEF2K-cKI, n = 9. p < 0.0001. (**D**) Percent of spontaneous alternations in the Y-maze task was decreased in eEF2K-cKI mice. % Alternation = (the number of correct alternations / total number of arm entries – 2) * 100. Cre, n = 9; eEF2K-cKI, n = 13. p < 0.0001. (**E**) Representative heatmaps for MWM. “P” indicates platform location. (**F**) Escape latency over 4 trials/day for 5 days of training in MWM. eEF2K-cKI mice had higher average escape latencies during the training phase. (**G**) Percent of time spent in target quadrant during MWM Probe trial was reduced in eEF2K-cKI mice. p < 0.0001. (**H**) Trending decrease in the number of platform crossings during MWM Probe trial in eEF2K-cKI mice. Cre, n = 11; eEF2K-cKI, n = 9. p = 0.0686 (**I**) Escape latency over 4 trials/day for 2 days of VP was not different between groups. Cre, n = 11; eEF2K-cKI, n = 9. Error bars represent ± SEM. ***p < 0.001, ****p < 0.0001; Student’s T-test.

Previous studies have indicated that eEF2K signaling may play an important role in learning and memory [16, 49–51]. We carried out a battery of behavioral assays to investigate the effects of eEF2K overexpression on learning and memory performance. We first evaluated long-term recognition memory using the novel object recognition (NOR) task where the mice distinguish between a familiar and novel object 24 h after familiarization [52, 53]. The Cre mice exhibited normal cognition and spent more time with the novel object with a positive discrimination index (Fig. 5A–C). In comparison, the eEF2K-cKI mice failed to distinguish the familiar object from the novel object with a significantly lower, negative discrimination index compared to the control group (Fig. 5A-C). eEF2K-cKI mice also spent less time interacting with either object compared to Cre controls (Supplemental Fig. 4C). Additionally, we performed the open field (OF) task to determine if there were any locomotor abnormalities and did not observe any differences between groups in distance and velocity moved (Supplemental Figs. 4A and B). These findings suggest impairments of recognition memory in the eEF2K-cKI mice.

Spatial working memory was evaluated in the Y-maze task. The eEF2K-cKI mice had a significantly lower percentage of correct arm alternations compared to Cre controls, suggesting impaired spatial working memory (Fig. 5D). Interestingly, the number of total entries was higher in the eEF2K-cKI mice (Supplemental Fig. 4D). We also assessed hippocampal-dependent spatial learning and memory using the hidden platform Morris Water Maze (MWM) task [53, 54]. During the training phase, the eEF2K-cKI mice took significantly longer to find the hidden platform compared to the Cre mice, suggesting impaired learning ability (Figs. 5E and 5F). The probe trial results showed that the eEF2K-cKI mice spent significantly less time in the target quadrant where the platform was located and had a trending decrease in the number of “platform” crossings compared to Cre controls (Figs. 5G and 5H). To determine potential memory-independent effects that may confound the above findings such as swimming ability and vision, we performed the visible platform (VP) task and found no difference between the Cre and eEF2K-cKI mice in the escape latency for the visible platform (Fig. 5I) or in the average swimming velocity (Supplemental Fig. 4E). These results indicate that overactive eEF2K signaling can impair learning and memory.

### Functional and structural synaptic deficits in eEF2K-cKI mice

**Fig. 6:**
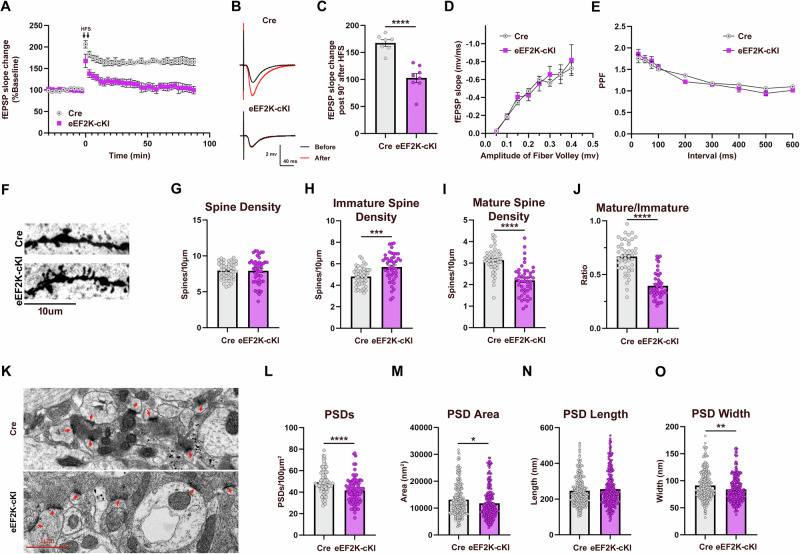
eEF2K overexpression leads to functional and structural synaptic deficits. (**A**) Acute hippocampal slices were stimulated with HFS to induce LTP. Arrows indicate HFS. eEF2K-cKI failed to show sustained LTP. Cre, n = 7 slices; eEF2K-cKI, n = 8 slices. (**B**) Representative fEPSP traces. (**C**) fEPSP slope 90 min after HFS stimulation was significantly decreased in the eEF2K-cKI mice. Cre, n = 7 slices; eEF2K-cKI, n = 8 slices. (**D**) Input/output curve assessing basal synaptic transmission was not significantly different between groups. Cre, n = 7 slices; eEF2K-cKI, n = 8 slices. p < 0.0001. (**E**) Curve of Paired-pulse ratio with varying interval time as a measure of presynaptic transmission was not different between groups. Cre, n = 7 slices; eEF2K-cKI, n = 8 slices. (**F**) Representative images from Golgi-Cox stain of CA1 dendritic spines. Original magnification x100. Scale bar = 10μm (**G**) Total spine density per 10μm was not significantly different between groups. p = 0.9800. (**H**) Immature spine density per 10μm was increased in eEF2K-cKI mice. Filopodia and thin spines were classified as immature. p = 0.0003. (**I**) Mature spine density per 10μm was decreased in eEF2K-cKI mice. Mushroom, stubby, and branched spines were classified as mature. p < 0.0001. (**J**) The ratio of mature to immature spines was reduced in eEF2K-cKI mice. n = 3 mice per group with 15 images per mouse, approximately 100μm of dendrite analyzed per image. p < 0.0001. (**K**) Representative TEM images of PSDs in CA1. Red arrows indicate PSDs. Scale bar = 1μm. Original magnification x9.8k. (**L**) eEF2K-cKI mice had fewer PSDs per an area of 100μm^2^. p < 0.0001. (**M**) Trending decrease in average PSD area in eEF2K-cKI mice. p = 0.0131. (**N**) PSD length was significantly increased in eEF2K-cKI mice. p = 0.2809. (**O**) PSD width was decreased in eEF2K-cKI mice. p = 0.0012. n = 4 mice per group with 15-20 images (ROIs) per mouse. Error bars represent ± SEM. *p < 0.05, **p < 0.01, ***p < 0.001, ****p < 0.0001; Student’s T-test.

Protein synthesis is essential for maintenance of long-term potentiation (LTP), a major form of synaptic plasticity and putative cellular basis of learning and memory [55]. Previous studies have shown that eEF2K/eEF2 signaling is associated with expression of plasticity-related proteins [56–58]. eEF2K is a calcium/calmodulin-dependent kinase, also known as CAMKIII, and is involved in multiple mechanisms of synaptic plasticity [56, 59]. LTP deficits have also been associated with neuropsychiatric disorders such as MDD and schizophrenia [60–63]. To determine if eEF2K overexpression affected synaptic plasticity, we performed ex vivo electrophysiology experiments to evaluate protein synthesis-dependent LTP at the CA3-CA1 synapses of acute hippocampal slices [64–66]. Compared to normal sustained LTP in the control Cre mice, the eEF2K-cKI mice exhibited LTP failure (Fig. 6A–C). Analysis of the synaptic responses with various stimulation intensities (input-output relationships) did not reveal any difference between the groups, indicating unaltered basal synaptic transmission in the eEF2K-cKI mice (Fig. 6D). We also investigated presynaptic function by measuring paired pulse facilitation (PPF), a form of short-term presynaptic plasticity [67, 68]. There was no significant difference in PPF between groups, suggesting normal presynaptic function (Fig. 6E). These findings demonstrate that synaptic plasticity is impaired with overactive eEF2K signaling.

The structural plasticity and dynamics of dendritic spines requires protein synthesis and is associated with synaptic plasticity and memory formation [69, 70]. Dendritic spine changes have also been associated with neuropsychiatric diseases [71–74]. We used the rapid Golgi-Cox stain approach [34] to assess dendritic spine density and morphology in apical dendrites in the CA1 region of the hippocampus based on published guidelines [75]. We did not observe any significant differences in total spine density between groups. However, further analysis revealed a significant increase in the number of immature spines (thin, filopodia), and a significant decrease in the number of mature spines (mushroom, stubby, branched) in the eEF2K-cKI mice compared to Cre controls (Fig. 6F-I). Accordingly, there was a decrease in the mature/immature spine ratio in the eEF2K-cKI mice compared to the control group (Fig. 6J). Using transmission electron microscopy (TEM) approach, we also evaluated postsynaptic densities (PSDs) which are specialized synaptic structures located on dendritic spines that are critical to synaptic function [76, 77]. We found that eEF2K-cKI mice had significantly reduced PSD density compared to Cre controls (Figs. 6K and 6L). Further morphological analysis revealed that PSDs of the eEF2K-cKI mice were significantly smaller and thinner than those in the Cre control mice (Fig. 6M-O). Taken together, overactive eEF2K signaling and eEF2 hyper-phosphorylation are associated with functional and structural synaptic deficits.

### Proteomic analysis of eEF2K-cKI mice

**Fig. 7:**
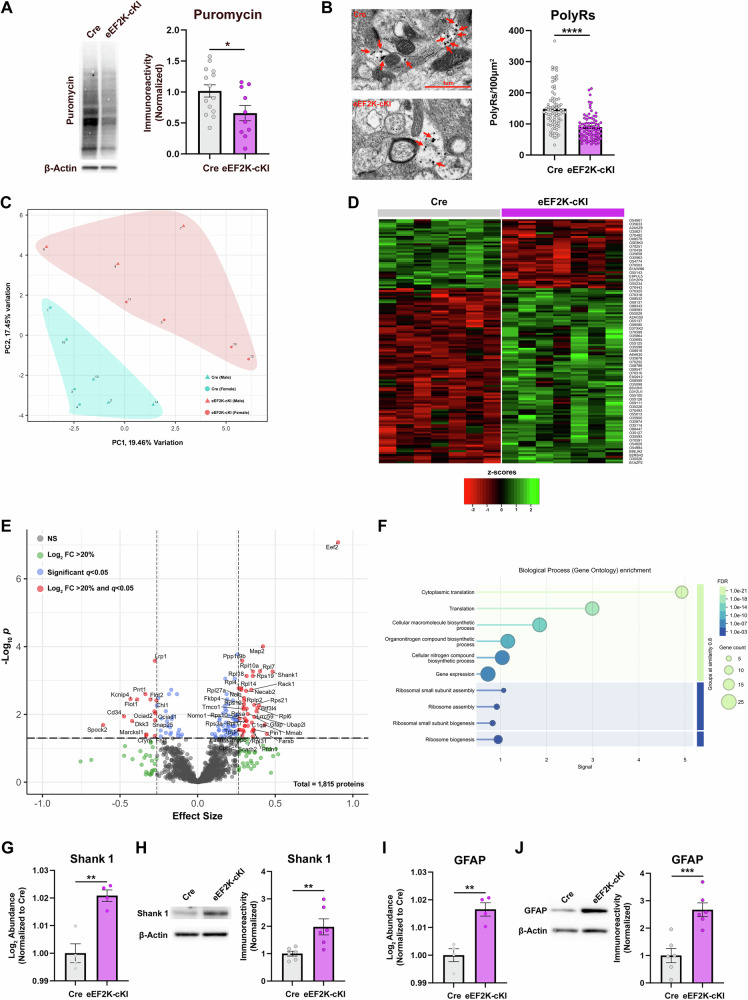
Altered proteome with eEF2K overexpression. (**A**) Western blot analysis of puromycin incorporation in the SUnSET assay to measure de novo protein synthesis shows decreased puromycin incorporation in the eEF2K-cKI mice. Cre, n = 14; eEF2K-cKI, n = 10. p = 0.0316. (**B**) Representative TEM images for CA1 polyribosomes near synapses. Polyribosomes within axons and somas were excluded from quantification. Red arrows indicate polyribosomes. Original magnification x9.8k. Scale bar = 1μm. eEF2K-cKI mice had fewer polyribosomes per an area of 100μm^2^. n = 4 mice per group with 15-20 images (ROIs) per mouse. p < 0.0001. (**C**) PCA plot of Cre and eEF2K-cKI mice used for proteomics. (**D**) Heatmap of the 133 proteins significantly dysregulated between the eEF2K-cKI and Cre mice. (**E**)Volcano plot showing the distribution of differently regulated proteins. Gray circles are the proteins that were not significantly altered. Green circles represent proteins that had a fold change greater that 20% but were not significant. Blue circles represent proteins that were significantly changed but had a fold change less than 20%. Red circles represent the proteins that were significant and had a fold change greater than 20%. (**F**) Gene ontology enrichment analysis of all 59 proteins that met the q-value and fold change requirements. n = 7 per group. (**G**) Proteomic analysis of the Log_2_ abundance of Shank1 normalized to Cre. n = 4 per group. p = 0.0020. (**H**) Western blot confirming an increase in Shank1 in eEF2K-cKI mice. n = 6 per group. p = 0.0097. (**I**) Proteomic analysis of the Log_2_ abundance of GFAP normalized to Cre. n = 4 per group. p = 0.0027. (**J**) Western blot confirming an increase in GFAP in eEF2K-cKI mice. n = 6 per group. p = 0.0009. Error bars represent ± SEM. **p < 0.01, ***p < 0.001; Student’s T-test.

Phosphorylation of eEF2 at T56 by eEF2K inhibits the elongation phase of protein synthesis. We assessed de novo protein synthesis using the surface sensing of translation (SUnSET) assay where acute hippocampal slices were incubated with a subthreshold dose of puromycin, a tRNA analogue, and then a western blot performed with an anti-puromycin antibody [78]. Levels of de novo protein synthesis, as indicated by puromycin measurement, were significantly decreased in eEF2K-cKI mice (Fig. 7A). Consistently, imaging analysis using TEM revealed that the eEF2K-cKI mice had fewer polyribosomes, which are sites of active translation [50, 53, 79], near synapses in the CA1 area of the hippocampus compared to Cre controls (Fig. 7B). These data demonstrate that eEF2K overexpression and subsequent eEF2 hyper-phosphorylation leads, as expected, to a decrease in de novo protein synthesis.

To determine how eEF2K-driven changes in protein synthesis affected the proteome, we performed mass spectrometry-based proteomics to determine changes in the hippocampal proteome associated with eEF2K overexpression. In brief, 16 age and sex matched samples underwent LC-MS/MS analysis in 2 batches. Two samples, one from each batch, were determined to be outliers via hierarchical clustering and were excluded from further analysis (Supplemental Fig. 5). The resulting 14 samples (n = 7 for each genotype) were corrected for batch effects using the sva package and principal component analysis shows that samples cluster based primarily on genotype (Fig. 7C) [80]. 1815 proteins were detected across 14 samples. After statistical analysis, we observed that 131 proteins were significantly changed and 59 met the criteria for a fold change greater than 20% with the expression pattern shown in a heatmap (Fig. 7D). Within this group, 44 proteins were significantly upregulated while 15 proteins were found to be downregulated as shown in Tables 1 and 2 and the volcano plot (Tables 1 and 2, Fig. 7E). To further determine the potential interactions and functions, GO enrichment analysis was performed. The majority of the proteins that were differentially expressed were associated with mRNA translation, gene expression, and ribosomal assembly and biogenesis (Fig. 7F). eEF2 showed the highest fold change among upregulated proteins. Of the upregulated proteins, 10 were 60 s ribosomal proteins and 8 were 40 s ribosomal proteins. Two proteins of interest that were significantly upregulated, Shank1 and GFAP, were verified via western blot (Fig. 7G-I). Additionally, several downregulated proteins were synaptic membrane components (Flot1, Flot2, SNAP25, Prrt1, and Marcksl1).

**Table 1:** List of upregulated proteins in hippocampus of eEF2K-cKI mice.

Accession	Protein Name	p-value	FDR	Fold Change
P58252	Elongation factor 2	4.64E-11	8.42E-08	0.905
D3YZU1	SH3 and multiple ankyrin repeat domains protein 1	2.4E-06	0.000546	0.483
Q9WUA2	Phenylalanine--tRNA ligase beta subunit	0.002301	0.037622	0.446
Q9D273	Corrinoid adenosyltransferase	0.000829	0.020343	0.432
P20357	Microtubule-associated protein 2	1.08E-07	9.84E-05	0.421
P14148	60S ribosomal protein L7	1.76E-06	0.000531	0.403
Q9CQH7	Transcription factor BTF3 homolog 4	0.000109	0.005517	0.396
P47911	60S ribosomal protein L6	0.000141	0.006551	0.386
Q922Q8	Leucine-rich repeat-containing protein 59	0.000199	0.008053	0.380
P03995	Glial fibrillary acidic protein	0.000361	0.011917	0.365
Q9CQR2	40S ribosomal protein S21	9.8E-05	0.005232	0.365
Q9CWM4	Prefoldin subunit 1	0.001577	0.029499	0.362
Q9QUR7	Peptidyl-prolyl cis-trans isomerase NIMA-interacting 1	0.001344	0.027729	0.358
P53026	60S ribosomal protein L10a	2.13E-06	0.000546	0.357
O35114	Lysosome membrane protein 2	0.003125	0.044664	0.356
Q9CZX8	40S ribosomal protein S19	3.65E-06	0.000737	0.355
P62900	60S ribosomal protein L31	0.0018	0.031959	0.352
P98086	Complement C1q subcomponent subunit A	0.000702	0.017448	0.349
P68040	Receptor of activated protein C kinase 1	1.74E-05	0.00188	0.346
Q9D898	Actin-related protein 2/3 complex subunit 5-like protein	0.000948	0.021779	0.324
Q80X50	Ubiquitin-associated protein 2-like	0.000262	0.009706	0.323
P97351	40S ribosomal protein S3a	0.000573	0.015519	0.316
P14206	40S ribosomal protein SA	0.00015	0.006785	0.316
P35980	60S ribosomal protein L18	4.07E-06	0.000739	0.316
Q91ZP9	N-terminal EF-hand calcium-binding protein 2	2E-05	0.002021	0.312
Q9CPR4	60S ribosomal protein L17	0.00059	0.015682	0.312
P25444	40S ribosomal protein S2	0.000967	0.021779	0.304
P63325	40S ribosomal protein S10	0.000431	0.013216	0.304
P63216	Guanine nucleotide-binding protein G(I)/G(S)/G(O) subunit gamma-3	0.000972	0.021779	0.303
P99027	60S acidic ribosomal protein P2	6.66E-05	0.003901	0.300
P14115	60S ribosomal protein L27a	4.33E-05	0.003295	0.299
P30416	Peptidyl-prolyl cis-trans isomerase FKBP4	8.89E-05	0.004968	0.299
Q8BGD5	Carnitine O-palmitoyltransferase 1, brain isoform	0.001853	0.032337	0.297
P63323	40S ribosomal protein S12	0.00013	0.006216	0.296
Q921L3	Calcium load-activated calcium channel	0.00016	0.006983	0.296
P62858	40S ribosomal protein S28	0.001559	0.029472	0.294
Q8BTZ7	Mannose-1-phosphate guanyltransferase beta	0.002748	0.041567	0.288
Q6R891	Neurabin-2	7.07E-07	0.000262	0.287
Q8VCM8	Nicalin	1.59E-05	0.00188	0.281
Q9CR57	60S ribosomal protein L14	1.31E-05	0.001695	0.281
O08914	Fatty-acid amide hydrolase 1	0.001496	0.028859	0.276
Q6GQT9	Nodal modulator 1	0.000438	0.013216	0.269
P63028	Translationally-controlled tumor protein	0.001387	0.028277	0.266
Q9D8E6	60S ribosomal protein L4	1.21E-05	0.001684	0.265

**Table 2:** List of downregulated proteins in hippocampus of eEF2K-cKI mice.

Accession	Protein Name	p-value	FDR	Fold Change
Q9ER58	Testican-2	0.000849	0.020551	-0.609
Q64314	Hematopoietic progenitor cell antigen CD34	0.000341	0.011446	-0.474
Q6PHZ8	Kv channel-interacting protein 4	4.88E-05	0.003539	-0.432
Q9QUN9	Dickkopf-related protein 3	0.000605	0.015688	-0.422
O08917	Flotillin-1	5.54E-05	0.003628	-0.390
O35449	Proline-rich transmembrane protein 1	2.82E-05	0.002526	-0.336
O54983	Ketimine reductase mu-crystallin	0.002835	0.042227	-0.332
P28667	MARCKS-related protein	0.002515	0.039019	-0.330
Q60634	Flotillin-2	5.33E-05	0.003628	-0.304
Q9D8W7	OCIA domain-containing protein 2	0.000207	0.008162	-0.278
P97447	Four and a half LIM domains protein 1	0.002922	0.043112	-0.276
Q91ZX7	Prolow-density lipoprotein receptor-related protein 1	7.21E-07	0.000262	-0.276
P60879	Synaptosomal-associated protein 25	0.000538	0.014894	-0.274
Q9CRD0	OCIA domain-containing protein 1	0.00024	0.009265	-0.272
P70232	Neural cell adhesion molecule L1-like protein	6.51E-05	0.003901	-0.266

Given the significant increase in GFAP and C1qa, we wanted to determine if there were glial changes associated with eEF2K overexpression. 60μm thick sections were stained with GFAP (green), Iba1 (red), and Dapi (blue). The eEF2K-cKI had significantly more GFAP positive cells compared to Cre controls while there was no significant difference in Iba1 positive cells (Supplemental Fig. 6). Additionally, changes in astrocyte morphology were observed with an increase in soma size with thicker processes and decreased ramification (Supplemental Fig. 6).

## Discussion

Overactive eEF2K signaling i.e. eEF2 hyper-phosphorylation has been implicated in neuronal diseases such as Alzheimer’s disease and depression [16, 31, 49, 50, 53]. Recent studies have shown that the anti-depressant drug, Ketamine, may act through the inhibition of eEF2K [6, 31]. Genetic deletion of eEF2K abolishes ketamine’s antidepressant effect suggesting that eEF2K hyperactivity may play a role in depression [6, 31]. Our lab has previously shown that eEF2 is hyperphosphorylated in the hippocampus of AD patients and in AD model mice [53]. Reducing eEF2 phosphorylation by genetic or pharmacologic means ameliorates learning and memory deficits in AD mouse models and improves synaptic function [49, 50]. This has led us to hypothesize that eEF2K over expression and hyper phosphorylation of eEF2 will cause depression-like behavior and cognitive deficits in mice.

We thoroughly and robustly characterized multiple neuropsychiatric-like behaviors by using domain specific z-scores. Using the z-score method of analysis allows for greater sensitivity and reliability in behavioral testing, especially when multiple behavioral paradigms are necessary to elucidate complex phenotypes like anxiety, depression, or apathy [81, 82]. This approach allows for the summary and comparison of multiple behavior tasks. Many of the behavior tests that we performed are commonly used in the study of depression [38]. However, we chose to separate these tests into multiple domains in order to better understand the role of eEF2K in these complex phenotypes. Additionally, while apathy, anxiety, and depression are often co-occurring, they are clinically separate and distinct disorders [83]. We also chose to separate depression-associated behaviors into multiple sub-domains depending on the specific aspect measured in the behavioral paradigm. For example, FST and TST were summarized as despair-like behavior while SPT was separated as anhedonia. We ultimately found that eEF2K overexpression resulted in a profound apathetic phenotype but did not lead to any significant changes in anxiety, despair, anhedonia, or social behaviors. Additionally, the effect of eEF2K on depressive phenotypes may only be visible under stress conditions. It may also be a question of acute vs. chronic eEF2K activation and future studies are needed to better understand this complex relationship.

### Apathy, dementia, and eEF2K

Apathy, defined by decreased motivation and goal-directed behavior, is often associated with dementia syndromes such as AD. Indeed, the most common NPS in AD patients is apathy, which causes greater caregiver burden, lower quality of life, faster cognitive and functional decline, and increased morbidity [84–92]. Apathy has also been associated with other diseases such as Parkinson’s disease, Huntington’s disease, amyotrophic lateral sclerosis, and geriatric depression [93–95]. Apathy commonly accompanies cognitive dysfunction and is a predictor of cognitive decline in many disease states [95–102]. Currently, little is known about the underlying mechanisms of apathy.

Historically, apathy is understudied with very few studies in preclinical models. There is currently no “gold standard” behavior test to measure apathy-like behavior. For our study, we chose 4 different rodent-typical behaviors that did not require training since eEF2K overexpression induces learning and memory deficits that could confound results. These tests (nestlet shredding, nest building, marble burying, and burrowing) were recently used to assess apathy-like behavior in mouse models of AD and down syndrome (DS) [37, 42]. Understanding the molecular underpinnings of apathy and other NPS is important to the treatment of AD and other neurological disorders. We demonstrate here, for the first time, that overactive eEF2K signaling is associated with an apathy-like phenotype. Such findings could provide insights into novel therapeutic strategies for treatment of NPS in AD and other neuronal diseases.

While eEF2K overexpression did not lead to a deficit in sociability, there was a deficit in the preference for social novelty. This decreased preference could be due to deficits in working memory as eEF2K-cKI displayed spatial working memory deficits in the Y-maze task. Additionally, several studies have found a connection between apathy and olfaction deficits which may contribute to the lack of preference for a novel mouse [103, 104].

### eEF2K overexpression is associated with synaptic and memory deficits

We first showed that eEF2K overexpression resulted in increased phosphorylation of eEF2 at T56. This phosphorylation site is phosphorylated by eEF2K and prevents eEF2 from binding to the ribosome [15]. Other phosphorylation sites on eEF2 may affect eEF2 activity by modulating T56 phosphorylation or dephosphorylation [105, 106]. The overall decrease in de novo protein synthesis agrees with the increased eEF2 phosphorylation at T56. Interestingly, total eEF2 was also increased in eEF2K-cKI mice. eEF2 mRNA contains 5’ terminal oligopyrimidine (TOP) motif [107]. TOP mRNAs have increased expression when eEF2 is phosphorylated [11]. Under conditions of cellular stress, when the rate of translation slows, the rate of TOP mRNAs translation remains constant leading to a relative increase in the proportion of TOP mRNAs [11]. This supports the findings of increase in total eEF2 in the eEF2K-cKI mice.

eEF2K signaling has also been linked to dendritic spine alterations [108]. Structural changes in dendritic spines are essential for normal neurodevelopment and brain function [72, 109]. Dendritic spine alterations also accompany activity changes and respond to synaptic plasticity mechanisms (LTP, LTD, etc.) [109–111]. They are also thought to underlie memory formation [112, 113]. Synaptic plasticity and these morphological changes are associated with protein synthesis and may involve eEF2K/eEF2 signaling [16, 59, 108]. We observed impairments in LTP, the process considered the cellular basis of learning and memory. LTP impairment was accompanied by changes in dendritic spine and PSD morphology. As mentioned, dendritic spines are closely linked to synaptic plasticity with mature spines (mushroom, branched, and stubby) usually considered “memory” spines [114]. Here we found a distinct decrease in the number of mushroom spines in eEF2K-cKI mice and a corresponding increase in the number of immature thin spines. eEF2K signaling controls expression of many plasticity-related proteins such as CAMKII, BDNF, and Arc, all of which have been implicated in synaptic plasticity and dendritic spine dynamics [59, 108, 115]. Increased eEF2K activity has previously been linked to learning and memory impairments [16, 51]. We found that eEF2K overexpression resulted in robust learning and memory impairments in the MWM, Y-maze, and NOR behavior paradigms. While dendritic spines and synaptic plasticity are most closely associated with learning and memory, evidence has accumulated for a significant role of dendritic spines in neuropsychiatric disorders as well [72, 116]. Dendritic spine changes have been linked to motivational impairments in schizophrenia [73, 74]. Dendritic spines and synaptic alterations have been implicated in the pathophysiology of MDD [61]. Further studies are needed to define the association between apathy-like behavior and dysregulated synaptic plasticity and changes in dendritic spine and synaptic morphology.

### Proteomic analysis of eEF2K overexpression

We investigated the effects of eEF2K overexpression in mature excitatory neurons on the proteome in the hippocampus. We performed mass spectrometry-based proteomics as well as gene ontology enrichment analysis. The majority of upregulated proteins were associated with translation and largely consisted of ribosomal proteins and translation factors. The subset of mRNAs whose translation is upregulated by eEF2K includes those with 5’ terminal oligopyrimidine (TOP) motif, many of which encode ribosomal proteins and translation factors, as seen in our results [11]. Additionally, eEF2 is an established TOP mRNA and was found to be upregulated in our proteomics data and this was further confirmed with western blot [107].

Several proteins that were differentially regulated are associated with dendritic spine morphology, synaptic function, and cognitive impairments. Shank1 is involved in dendritic spine morphology and has been associated with cognitive deficits [117, 118]. In schizophrenia, decreased Map2 has been associated with fewer small dendritic spines [119]. In concordance, our data suggests that increased Map2 may be associated with increased numbers of small dendritic spines. Neurabin-2, also known as spinophilin, is a synaptic scaffolding protein that has been implicated in dendritic spine morphology and synaptic plasticity [120, 121]. Additionally, Chl1 is a cell adhesion molecule that has been associated with neurite outgrowth and synaptic plasticity [122].

A number of upregulated proteins in eEF2K-cKI mice have been linked to neuropsychiatric and neurodegenerative diseases that are often accompanied by apathy symptoms. Shank1 is a synaptic scaffold protein that interacts with the PSD and has been implicated in neuropsychiatric and neurodegenerative disorders such as autism spectrum disorder, schizophrenia, and AD [123, 124]. Additionally, Shank1 hypomethylation was associated with depressive episodes in adolescents [125]. Map2 is a neuronal cytoskeleton protein that has been implicated in schizophrenia, AD, and mood disorders [126, 127]. FAAH is an endocannabinoid hydrolase and its upregulation is implicated in Major depressive disorder (MDD), AD, and schizophrenia [128–130]. Neurabin-2 has also been linked to schizophrenia [131]. Another protein of interest is SNAP25, a SNARE complex member. SNAP25 is decreased in patients with mood disorders and schizophrenia [132, 133]. GFAP is an inflammatory marker that has been implicated in numerous neuropsychiatric and neurodegenerative disorders. Increased GFAP has been proposed as a useful biomarker for AD [134]. GFAP levels are negatively correlated with cognitive measures in AD and increased GFAP is associated with worse cognition [135, 136]. MDD and cognitive decline was associated with higher GFAP levels in older adults [137]. However, GFAP expression in MDD is somewhat mixed with some studies finding increased GFAP or no change in GFAP, while many show decreased GFAP expression [138, 139]. Similarly, GFAP may be increased in schizophrenia, but many studies show different results [138]. Furthermore, several studies have correlated increased GFAP with apathy [140–142]. Increased GFAP may explain the robust apathy-like phenotype with a lack of despair-like behavior. More work is needed to determine a direct relationship between these implicated proteins, eEF2K signaling, and disease pathogenesis.

It is important to note that proteomics method in the current study does not account for nascent changes in the proteome. Multiple studies have shown that changes in elongation rate affect the specific mRNAs being translated [143, 144]. eEF2 is directly responsible for the translocation of the ribosome down the mRNA [14, 15]. Phosphorylation of eEF2 may affect the rate of cap-dependent translation of certain mRNAs while also promoting the cap-independent translation of TOP mRNAs. In this way, it is possible that there are differences in the nascent as well as the total proteome. Further studies using alternative approaches to determine how p-eEF2 upregulation affects the nascent proteome are warranted.

In conjunction with the proteomic analysis, immunofluorescence staining revealed an increase in GFAP+ cells with altered morphology in the eEF2K-cKI mice. eEF2K overexpression lead to more astrocytes as well as more reactive astrocytes compared to controls. This is interesting and could be a response of glia to neuronal stress. Given the decrease in the number of PSDs, this might also suggest an increase in synaptic engulfment in eEF2K-cKI mice. Further studies are needed to determine the underlying mechanism of neuronal eEF2K expression and glial expression and reactivity.

Lastly, this study focused on the thorough characterization of hippocampal changes and their association with neuropsychiatric behaviors. Several studies have shown that the hippocampus, while most widely known for its role in learning and memory, is also involved in emotional regulation and has been implicated in mood disorders [145–147]. Apathy has also been associated with decreased hippocampal volume [148]. The hippocampus is also connected to other emotion-related brain regions such as the anterior cingulate cortex (ACC) and the amygdala [63]. However, the prefrontal cortex and other brain regions, such as the ACC, are also known to play a role in apathy and mood disorders [149]. Future work is necessary to determine how altered protein synthesis and synaptic function in different brain regions contribute to behavioral phenotypes and apathy.

In summary, overexpression of eEF2K resulted in dysregulation of multiple proteins that are known to be involved in neuropsychiatric and neurodegenerative disorders which highlights the potential critical role of eEF2 hyper-phosphorylation in their pathogenesis.

eEF2K has recently been studied for its contributions to AD pathogenesis and its potential role in MDD and other neuropsychiatric disorders. In this study, we found that contrary to our hypothesis based on eEF2K’s role in the rapid anti-depressant action of ketamine, eEF2K overexpression did not result in despair-like behavior. However, we determined a novel role of eEF2K in apathy-like behavior, which is common to many neurodegenerative and neuropsychiatric disorders. In addition, we demonstrated that eEF2K overexpression and subsequent hyperphosphorylation of eEF2 lead to a marked memory deficit. This was accompanied by impaired synaptic plasticity and structural synaptic changes. In summary, eEF2K may play a larger role in apathy-like behavior and learning and memory impairments. More work is needed to better understand these complex relationships.

## Materials and methods

### Mice

All mice were housed at the Wake Forest School of Medicine barrier facility under the supervision of Institutional Animal Care and Use Committee. The facility operates in accordance with standards and policies of the US Department of Agriculture’s Animal Welfare Information Center (AWIC) and the NIH Guide for Care and Use of Laboratory Animals. Mice adhered to a 12-hour light/12-hour dark cycle, with regular feeding, cage cleaning, and 24-hour food and water access. All genotyping was done by polymerase chain reaction (PCR).

Rosa26-CAG-LoxP-STOP-LoxP-Eef2k knock-in mice were generated by the University of North Carolina at Chapel Hill Animal Models Core using CRISPR/Cas9-mediated genome editing. Mice were bred with CAMKII-cre mice [150] to produce eEF2K conditional overexpression (eEF2K-ckI) mice and cre control littermates. Male and female mice 3-5 months old were used for all experiments. For each behavioral experiment, multiple cohorts of mice were used. Mice with significant injury or evidence of seizure activity were excluded from analysis. No randomization was used in determining groups.

### RNA isolation and quantitative RT-PCR

The hippocampus was dissected out in ice cold PBS and total RNA was isolated with Trizol (Invitrogen, catalog #15596026). The first strand cDNA was generated using qScript cDNA SuperMix (Quanta bio, catalog #95048-025). The genes were amplified using quantitative PCR with PerfeCTa SYBR Green FastMix (Quanta bio, catalog #95072-250). The following PCR protocol was used: 95 °C for 2 min, then 40 cycles of 95 °C for 10 s, 60 °C for 30 s. To determine relative gene expression, ^ΔΔ^Ct was calculated. GAPDH was used as the normalization control. Forward eEF2K primer sequence: 5’- CCACTTGGAGCACTACATTGAGG -3’. Reverse eEF2K primer sequence: 5’- ATAAAGGTCACCCACACCCTGG -3’.

### Western Blot

Mouse hippocampal tissue was dissected and flash frozen on dry ice. Lysis and gel electrophoresis were performed as previously described [151]. Briefly, the tissue was sonicated in lysis buffer with protease and phosphatase inhibitors. Protein quantification was determined using the Bradford colorimetric Assay (Thermo Fischer Scientific, catalog #23227). Equal amounts of protein lysate from each sample were loaded on 4 - 12% Tris-glycine SDS-PAGE gels (Bio-Rad 18-well gel, catalog #5670184) followed by standard gel electrophoresis. After transfer, membranes were blocked for 10 min with SuperBlock TBS Blocking Buffer (Thermo Fischer Scientific, catalog #37535). All primary and secondary antibodies were diluted in 5% milk/TBST or 5% BSA/TBST. Blots were incubated with the following primary antibodies overnight at 4 °C: eEF2K (1:500, Cell Signaling Technology, catalog 3692), phospho-eEF2 (Thr56) (1:1000, Cell Signaling Technology, catalog 2331); eEF2 (1:1000, Cell Signaling Technology, catalog 2332); β-Actin (1:5000, Sigma Aldrich, catalog A2228); GFAP (1:1000, Cell Signaling Technology, catalog 12389S); Shank1 (1:500, Santa Cruz, catalog sc-398630). Blots were then incubated in secondary antibodies for 2 h at room temperature. The ChemiDoc Imaging System (Bio-Rad) was used for protein visualization. Densitometric analysis was performed using ImageJ software (NIH). Phospho-proteins were normalized to the levels of total protein; total proteins were normalized to the housekeeping protein β-Actin.

### Acute Hippocampal Slice Preparation

A Leica VT200S vibratome was used to prepare 400 μm transverse acute hippocampal slices as previously described [64]. Slices were maintained in ACSF bubbled with 95% O_2_ / 5% CO_2_ at room temperature for 2 hr before experiment. ACSF contained the following: 118 mM NaCl, 3.5 mM KCl, 2.5 mM CaCl2, 1.3 mM MgSO4, 1.25 mM NaH2PO4, and 15 mM glucose.

### Surface Sensing of Translation (SUnSET) Assay

Surface Sensing of translation was performed as previously described [53, 78]. Briefly, acute hippocampal slices were incubated in 1 μg/mL puromycin for 1 hr at 32 °C in bubbling ACSF. Slices were flash frozen on dry ice, lysed, and standard gel electrophoresis run. The puromycin-labeled proteins were identified by antibody (1:10,000, Millipore, catalog #MABE343). Total lane density was used to determine protein synthesis levels and densitometry was done using ImageJ (NIH).

### Transmission Electron Microscopy (TEM)

Electron microscopy was performed as previously described [53]. Briefly, CA1 was dissected from acute 1 mm transverse hippocampal slices and fixed in 1% PFA + 2.5% glutaraldehyde in 0.1 M Millonig’s phosphate buffer (pH 7.3) overnight. Samples were washed and postfixed in 1% osmium tetroxide in PBS for 1 hr. Samples were then dehydrated through a graded ethanol series and incubated in propylene oxide twice for 15 min each. Samples were infiltrated with Spurr’s resin and cured overnight at 70 °C. 90 nm sections were made using a Reichert-Jung Ultracut E Ultramicrotome. Sections were stained with lead citrate and uranyl acetate. Samples were imaged on FEI Tecnai Bio Twin Transmission Electron Microscope (120 keV) with a 2 Vu AMT camera at x9.8k magnification. 4 mice were used per genotype and 15-20 images of the stratum radiatum of area CA1 were taken. ImageJ was used to analyze images and analysis was performed blinded.

### Electrophysiology

Acute hippocampal slices were prepared as described above. Following the 2 h incubation period, slices were maintained at 32 °C, and monophasic current stimuli of 100μs were delivered with a bipolar silver electrode in the stratum radiatum of area CA3. Field excitatory postsynaptic potentials (fEPSPs) were recorded using a glass microelectrode from the stratum radiatum of CA1. LTP was induced using a high-frequency stimulation (HFS) comprised of two 1 s, 100 Hz trains, with a 60 s interval, delivered at 60-70% of evoked spike intensity.

### Golgi Staining

The Rapid Golgi Kit (FD NeuroTechnologies, catalog PK401) according to the manufacturer’s instructions. Transverse 100μm hippocampal slices were made after impregnation with a Leica VT1200S vibratome and mounted on gelatin-coated slides. Sections were developed and fixed according to kit instructions. Imaging was performed with a Keyence BZ-X710 All-in-one Fluorescent Microscope with a x100/NA 1.45 oil immersion lens. 5 images within the stratum radiatum of area CA1 were taken per each hippocampal slice. Spines were classified based on published guidelines [75]. Imaging and spine analysis were performed blinded.

### Behavioral Assays

For all behavioral assays, mice were handled prior to behavioral testing and were habituated to the testing room for 1 hr before start of experiments. Experiments were performed during the 12-hr light cycle. Behavioral assays were ordered from least stressful to most stressful. Experimenter was blinded to all genotypes during experiments and analysis.

### Morris Water Maze (MWM)

A 5-day MWM protocol was performed with 4 trials a day (60 s maximum per trial, 15 min interval between trials) as previously described [53]. Escape latency was measured for each trial. Two hours after training on the 5^th^ day, a probe trial was performed. EthoVision XT Tracking Software (Noldus Information Technology) was used to track trajectories, time spent in maze quadrants, distance and velocity.

### Visible Platform (VP)

VP was performed after MWM with a 2-day protocol consisting of 4 trials a day (60 s maximum per trial, 15 min interval between trials). The platform was marked by a visual cue and moved randomly among 4 locations. Escape Latency was measured for each trial.

### Novel Object Recognition (NOR)

Mice were habituated to the experiment chamber 1 day before start of experiments. A 2-day familiarization protocol was used. On the first 2 days, mice were placed in a white plastic chamber (40 cm x 40 cm x 40 cm) with 2 identical objects and allowed to explore for 5 min. Twenty-four hours after familiarization, mice were tested in the chamber with one object replaced with a novel object and allowed to explore for 5 min. Objects were randomly assigned to each mouse and location of the novel object was counterbalanced. Time spent exploring objects was measured manually and by EthoVision XT Tracking Software (Noldus Information Technology). Mice with <10 s total interaction time were excluded from analysis. Discrimination index was calculated as the novel object interaction time minus the familiar object interaction time divided by the total interaction time.

### Y-maze

Y-maze as a measure of spatial working memory was performed as previously described [152]. The Y-maze apparatus consisted of 3 opaque arms of equal length at 120° angles from each other. The mouse was placed in the center of the maze and allowed to explore freely for 8 min. The entries into each arm, defined as all four limbs within an arm, were recorded. An alternation was defined as consecutive entries into all three arms. Percent alternation was calculated as (the number of alternations / total number of arm entries – 2) * 100. High percent alternation is indicative of good spatial working memory.

### Nestlet Shredding (NS)

All mice were habituated in the testing room for 1 hour prior to start of testing. Group-housed mice were placed individually into a clean mouse cage with bedding and one piece of cotton fiber nestlet (5 cm×5 cm, 5 mm thick) that was pre-weighed and placed on top of the bedding in each cage. Each mouse was left undisturbed in the cage with the nestlet for 30 minutes. After the test, the nestlet was left in open space overnight to dry and was weighed again. The last weight and the starting weight were used to calculate percentage of nestlet shredded, a smaller number will indicate more severe apathy-like behavior [153].

### Nest Building (NB)

Nest building was performed after the nestlet shredding and marble burying tests. The group-housed mice were habituated with a cotton fiber nestlet (5 cm×5 cm, 5 mm thick) in their home-cage overnight before the day of testing. On the test day, mice were individually housed in a new cage with fresh, unscented bedding with a piece of clean and pre-weighed nestlet. The mice were allowed to behave freely overnight, and the nests were evaluated the following morning. The nest from each mouse was photographed and the nests were graded using scores ranging from 1 (very poor/no nest building) to 5 (optimal nest building) with half-point scores for nests falling between categories according to a previously published scoring scheme by Deacon [43]. Similar to the nestlet shredding test, the amount of nestlet left unshredded was weighed and used to calculate the percentage of unshredded nestlet. A lower nest score and a higher percentage of unshredded nestlet indicates more severe apathy-like behavior.

### Marble Burying Test (MBT)

Group-housed Mice were habituated in the testing room 1 hour before the. A standard rat cage (26 cm×48 cm x 20 cm) was used with 5 cm layer of unscented mouse bedding material. 15 standard glass toy marbles (assorted styles and colors/15 mm diameter, 5.2 g in weight) were gently put on the surface of the bedding in 3 rows of 5 marbles. Each mouse was gently placed in the cage in a corner away from marbles and allowed to behave freely, undisturbed for 30 minutes. The percent of total marble volume buried for each marble was recorded and the number of those with > 2/3 total volume buried were counted as buried. Fewer marbles buried indicates more severe apathy-like behavior [153].

### Burrowing

Group-housed mice were habituated with a burrowing tube filled with food pellets in their home-cage overnight before the day of testing. On the testing day, the mice were individually housed in a new cage with fresh bedding. A clean burrowing tube was filled with 200 grams of food pellets and placed in each cage. For the 2-hour burrowing test, the filled burrowing tube was placed in the cage around 2:00-3:00 pm and the mouse was allowed to behave freely for two hours. Following the 2-hour period, the amount of food left in the tube was weighed and used to calculate the amount burrowed. For the overnight burrowing test, the burrow tube was emptied and refilled with 200 g of new food pellets and placed back in the cage overnight. On the following morning, the amount of food left in the tube was weighed and used to calculate the amount burrowed [154, 155]. Smaller amount burrowed will indicate greater apathy-like behavior.

### Open Field (OF)

Mice were placed in a white plastic chamber (40 cm x 40 cm x 40 cm) and allowed to explore for 15 min. Time spent in the center and periphery of the chamber, as well as distance moved, and average velocity was measured using EthoVision XT Tracking Software (Noldus Information Technology). The percentage of total time spent in the periphery of the chamber was calculated.

### Novelty-Induced Hypophagia (NIH)

NIH was performed as previously cited with minor adjustments [48]. Mice were trained to drink vanilla Ensure® in their home cage over the course of three days for 30 min each day. The Ensure® was presented in a 50 mL conical tube with a rubber stopper through the wire rack of the cage lid. The amount consumed was weighed in grams and the latency to drink was recorded for each training day. Following training, mice underwent home cage testing following the same procedure as during training. Training and home cage testing were performed in low light. The day after home cage testing, mice underwent novel cage testing where the mice were presented the ensure in a novel cage without a wire rack or bedding and in bright light conditions. The amount consumed was weighed in grams and the latency to drink was recorded for both testing days.

### Novelty-Suppressed Feeding Test (NSFT)

NSFT was performed as previously described with minor adjustments [40]. Mice were food deprived for 48 h prior to behavioral testing with a 2-hour free feeding period after the first 24 h. Following food deprivation, mice were placed in a white plastic chamber (40 cm x 40 cm x 40 cm) that had 2 cm of bedding in the bottom and a food pellet position in the middle of the arena and secured to a platform at the level of the bedding. The latency for the mouse to begin eating the food pellet was recorded. The mouse was then immediately returned to its home cage with a pre-weighed food pellet for 5 min. the weight of food consumed after 5 min was recorded.

### Sucrose Preference Test (SPT)

SPT was performed with minor adaptations as previously described [40]. Mice were first habituated to two bottles of water in their home cage overnight. Water was presented in a 50 mL conical tube fitted with a rubber stopper on the left and right side of the cage. The amount of water consumed was weighed after 24-hour access. One bottle was replaced with a 1% (w/v) sucrose solution. The position of the sucrose solution was randomly assigned for each mouse. The amount of sucrose consumed was weighed after 24-hour access. This was repeated for 2 more days, and the position of the sucrose solution was alternated each consecutive day.

### Forced Swim Test (FST)

Mice were placed in a 4-L glass beaker (Corning Pyrex 1000-4 L) containing 3 L of 24  °C water (75% full) [31]. They were recorded at the level of the animal horizontally for 6 minutes. The final 4 minutes were used for scoring immobility as described in literature. The videos from FST were scored using DBscorerV2 which is an automatic mobility based quantification software [39]. We used the recommended threshold of 1.6. Results were compared to blinded hand scored results and found to be accurate.

### Tail Suspension Test (TST)

Mice were suspended by their tail in a custom-designed white chamber [31]. Seventeen cm of tape was attached at the end of their tail with 1 cm of tail exposed. The tape was then wrapped around the chamber to suspend the mouse with 12 cm of slack. Mice were recorded for 6 minutes and the entire video was used for scoring using DBscorerV2 with the recommended threshold of 0.6 [39]. Results were compared to blinded hand scored results and found to be accurate.

### 3-Chamber Sociability Test (3-CST)

Protocol for 3-chamber sociability test was adapted from Yang, et al. [45]. Stranger mice were habituated in wire cups prior to behavioral testing. Multiple stranger mice were used and alternated between experimental mice so that each stranger mouse did not spend significant amounts of time in the wire cup. The experiment mouse was first habituated for 5 min in the middle compartment of the 3-chamber apparatus with the doors closed. Immediately following, empty wire cups were placed in the left and right-side chambers, doors were opened, and experiment mouse was allowed to explore all three chambers and cups for another 5 min. After the 5 min, the experiment mouse was placed back in the middle chamber with the doors closed while the stranger 1 mouse was placed in a cup. The side for stranger mouse was determined to be the opposite side of the chamber the experiment mouse was last in. The doors were opened and the experiment mouse was allowed to explore for 10 min. The experiment mouse was then placed back in the middle compartment with the doors closed while the stranger 2 mouse was added to the empty wire cup on the opposite side of the stranger 1 mouse. The doors were opened and the experiment mouse was allowed to explore for another 10 min. The time the experiment mouse spent interacting with each cup during each 10 min session was measured.

### Composite z-score Analysis

Z-scores were calculated as previously described for each behavior test independently for each mouse relative to the mean and standard deviation (SD) of Cre controls so that changes of ± 1 z-score unit represented ± 1 SD of the control sample’s behavior [37]. To reduce the variance and enhance the reliability of our data, we further created composite scores (i.e., a combined z-score for each mouse across all tests) for 4 different behavioral domains: anxiety-like behavior, apathy-like behavior, despair-like behavior, and anhedonia-like behaviors. The raw z-scores were corrected so that a higher value was indicative of greater anxiety, apathy, despair, or anhedonia, respectively. The composite score for anxiety-like behaviors was calculated using the following equation: (z_*i*_(OF) + z_*i*_(NIH Latency) + z_*i*_(NIH Consumption) + z_*i*_(NSFT))/4, which will indicate overall anxiety-like behavior severity. The composite score for apathy-like behaviors was calculated using the following equation: (z_*i*_(NS) + z_*i*_(MB) + z_*i*_(NB % unshredded) + z_*i*_(NB score) + z_*i*_(2 hr burrowing) + z_*i*_(overnight burrowing))/6, which will indicate overall apathy-like behavior severity [42, 81]. The composite score for despair-like behaviors was calculated using the following equation: (z_*i*_(FST Immobility) + z_*i*_(FST Latency) + z_*i*_(TST Immobility) + z_*i*_(TST Latency))/4, which will indicate overall despair-like behavior severity. The score for anhedonia-like behavior was comprised of the individual z-score for sucrose preference.

### Proteomics

Mouse hippocampi were dissected and flash frozen on dry ice. Tissue was lysed in PBS with 2x protease inhibitor using a bead mill homogenizer. Lysed tissue was incubated in 2x RIPA buffer on ice for 30 min. Samples were centrifuged at 18,000 x g for 10 min and supernatant collected and protein quantification assay performed. 10 mM dithiothreitol (DTT) was added to 50 μg of protein and incubated at 55 °C for 30 min. 30 mM iodoacetamide (IAM) was added and samples incubated in the dark at room temperature for 30 min. 4x sample volume of cold acetone was added and samples incubated overnight at -20 °C. Samples were centrifuged at 14,000 x g for 10 min at 4 °C and supernatant discarded. Pellet was suspended in 100 μL of 50 mM ammonium bicarbonate. Samples were incubated with 1.0 μg of trypsin/lys-C mixture overnight at 37 °C. Digestion was quenched by adding formic acid (1.0% w/w final) and the mixture was purified using C18 tip. Peptides were prepared in 100 μL of 5% acetonitrile with 1% formic acid for LC-MS/MS analysis.

Samples were analyzed on a LC-MS/MS system consisting of an Orbitrap Eclipse Mass Spectrometer (Thermo Scientific, Waltham, MA) and a Vanquish Neo nano-UPLC system (Thermo Scientific, Waltham, MA). Peptides were separated on a DNV PepMap Neo (1500 bar, 75 μm x 500 mm) column for 120 min employing linear gradient elution consisted of water (A) and 80% acetonitrile (B) both of which contained 0.1% formic acid. Data were acquired by top speed data dependent mode where maximum MS/MS scans were acquired per cycle time of 3 seconds between adjacent survey spectra. Dynamic exclusion option was enabled which duration was set to 120 seconds. To identify proteins, spectra were searched against the UniProt mouse protein FASTA database (17,082 annotated entries, Oct 2021) using the Sequest HT search engine with the Proteome Discoverer v2.5 (Thermo Scientific, Waltham, MA). Search parameters were as follows: FT-trap instrument; parent mass error tolerance, 10 ppm; fragment mass error tolerance, 0.6 Da (monoisotopic); enzyme, trypsin (full); # maximum missed cleavages, 2; variable modification, +15.995 Da (oxidation) on methionine; static modification, +57.021 Da (carbamidomethyl) on cysteine.

In 2 batches, 16 age and sex matched samples (n = 8 per genotype) underwent LC-MS/MS analysis. Two samples, one from each batch, were determined to be outliers via hierarchical clustering and were excluded from further analysis. The resulting 14 samples (n = 7 for each genotype) were corrected for batch effects using the sva package [80]. Principle component analysis was performed using PCAtools package [156]. Normalized abundances were analyzed for all samples using R (version 4.5.0), excluding proteins with missing values. A 2-tailed t-test was performed for each protein reported and FDR-corrected p-values were used to determine significantly changed proteins. Proteins that had a q-value < 0.05 and a fold change greater than 20% were reported. Gene ontology enrichment analysis was performed on all proteins that met the above criteria.

### Immunofluorescence

Mice were anesthetized using Avertin (Sigma-Aldrich #T4, 840-2) intraperitoneal injection at a dose of 0.80 g per gram of body weight. Mice were transcardially perfused with 15 mL of cold PBS then perfused with 50 mL of cold 4% paraformaldehyde (PFA) at a 5 mL/min flow rate. The brain was dissected out and fixed overnight in cold 4% PFA in PBS. Half brain was subsectioned to 60 μm using a Leica VT1200S vibratome and washed twice in 0.02% Triton X-100 in PBS for 10 min each. Free-floating sections were then permeabilized in 300 mM Glycine and 10% DMSO, in 0.02% Triton X-100 in PBS for 1.5 h. Slices were washed twice in 0.02% Triton X-100 in PBS for 5 min each. Slices were blocked for 2 h in 6% normal goat serum in 0.02% Triton X-100 in PBS. Slices were washed twice in 0.01% Triton X-100 in PBS for 5 min each. Slices were then additionally blocked against endogenous IgG with Fab Fragment Goat Anti-Mouse IgG (1:50, Jackson ImmunoResearch, 115-007-003) in 0.01% Triton X-100 in PBS at 4 °C overnight on a shaker. Slices were washed 3 times for 15 min in 0.2% Tween-20 in PBS with 0.001 mg/mL Heparin (PTwH). Slices were then incubated for 2 days at 4 °C in primary antibodies: GFAP (1:500, Cell Signaling Technology, catalog 12389) and Iba1 (1:500, Abcam, ab178846) in 0.5x PTwH with 3% normal goat serum. Slices were washed with 0.5xPTwH 4 times for 20 min. Slices were then incubated for 36-48 h at 4 °C in secondary antiobdies: Goat anti-Mouse IgG Alexa Fluor® 488 conjugate (1:500, Invitrogen, A11001) and Goat anti-Rabbit IgG Alexa Fluor® 568 conjugate (1:500, Invitrogen, A-11011) in 0.5x PTwH with 3% normal goat serum. Sections were washed 3 times for 20 min in 0.5x PTwH then another 3 times for 20 min in PBS. Sections were incubated in Dapi (Sigma-Aldrich, MBD0015) for 4 h. Sections were washed 3 times with PBS for 30 min then mounted with Vectashield anti-fade mounting medium (Vector Laboratories, H-1000-10). The sections were then imaged using an Olympus FV1200 confocal microscope. All parameters (pinhole, contrast, gain, and offset) were held constant for all sections across the same experiment. Images were analyzed using ImageJ software (NIH).

### Statistical analysis

Data are presented as mean ± SEM. For comparisons between two groups, a two-tailed unpaired Student’s *t*-test was used. For significant differences in variance, Welch’s correction was used. When applicable, two-way ANOVA was used followed by individual post hoc tests. Error probabilities of *p* < 0.05 were considered statistically significant. Sample size was chosen following previous publications. Variance was similar between the groups that were being statistically compared based on our observation. Data were analyzed using GraphPad Prism software.

### Ethics approval

All methods and protocols involving animals were approved by the Institutional Animal Care and Use Committee (IACUC) of Wake Forest University School of Medicine (A24-094). Mice were kept in compliance with the NIH Guide for the Care and Use of Laboratory Animals (National Academies Press, 2011).

## Supplementary information

Supplemental Figures

## Data Availability

All data are available upon request.
