# Supplementary material for: Overactive Neuronal eEF2K/eEF2 signaling is associated with cognitive impairment and apathy-like behavior
Source: Mol Psychiatry. 2025 Dec 14;31(5):2454–71. doi: 10.1038/s41380-025-03408-z (PMC13099653; doi:10.1038/s41380-025-03408-z)
Supplement: Supplementary file 1 — Supplemental Figures [file 41380_2025_3408_MOESM1_ESM.docx]

**Supplemental Figures**

**
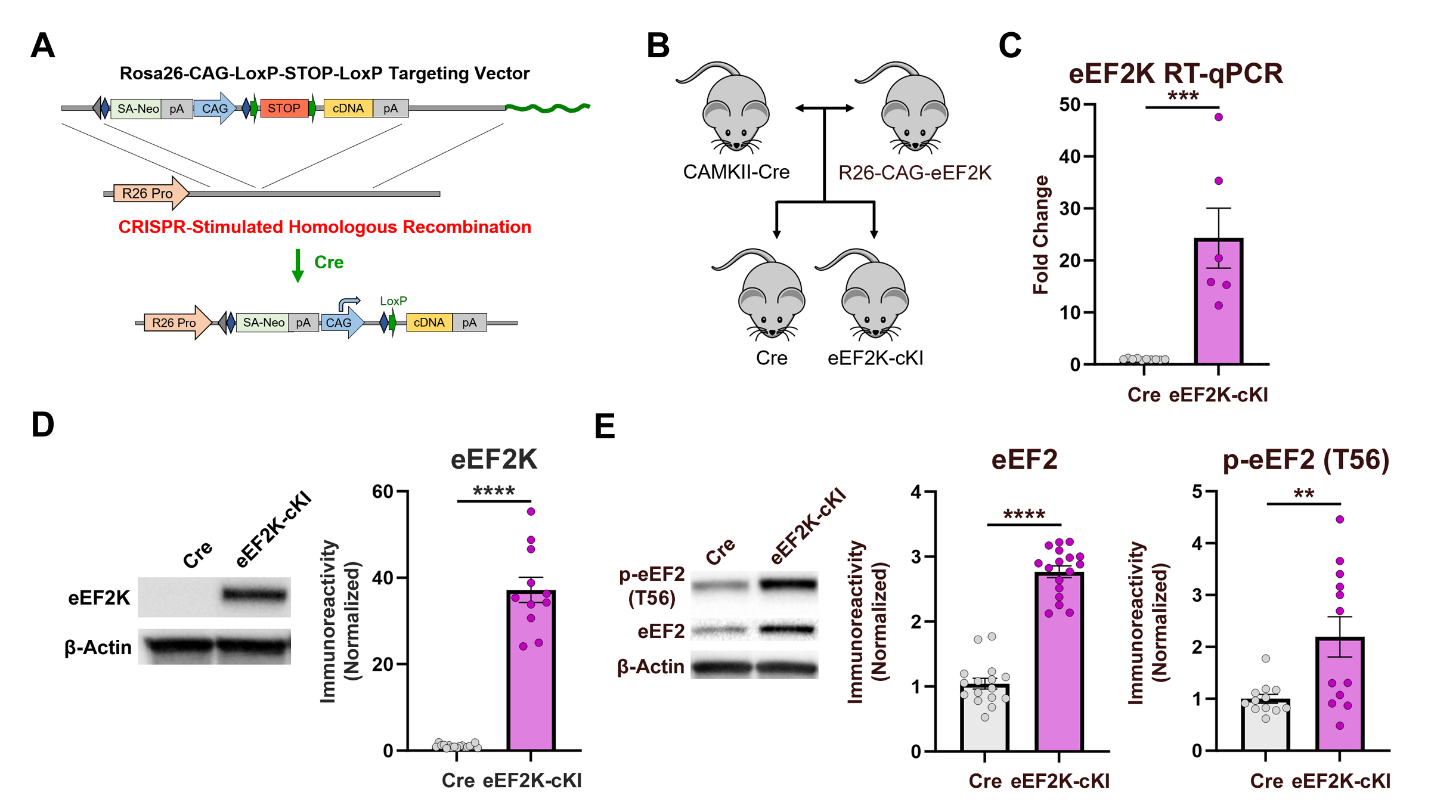
**

***Supplemental Figure 1: eEF2K overexpression leads to increased eEF2 phosphorylation.*** (**A**) Schematic of targeting vector with eEF2K cDNA and the CRISPR stimulated homologous recombination of the targeting vector into the Rosa26 gene. (**B**) Breeding schematic for the eEF2K-cKI mice and Cre littermates. (**C**) Quantitative RT-PCR showing the fold change in eEF2K mRNA in the eEF2K-cKI mice normalized to Cre. Cre, n=10; eEF2K-cKI, n=6. p=0.0001. (**D**) Western blot analysis of eEF2K in the hippocampus shows increased eEF2K protein expression in eEF2K-cKI mice. eEF2K normalized to β-Actin. Cre, n=17; eEF2K-cKI, n=11. p<0.0001. (**E**) Western blot analysis of p-eEF2 in the hippocampus shows increased p-eEF2 (T56) in eEF2K-cKI mice. eEF2 normalized to β-Actin; p-eEF2 normalized to eEF2. Cre, n=12; eEF2K-cKI, n=12. eEF2 p<0.0001; p-eEF2 p=0.0065. Error bars represent ± SEM. **p<0.01, ***p<0.001, ****p<0.0001; Student’s T-test.


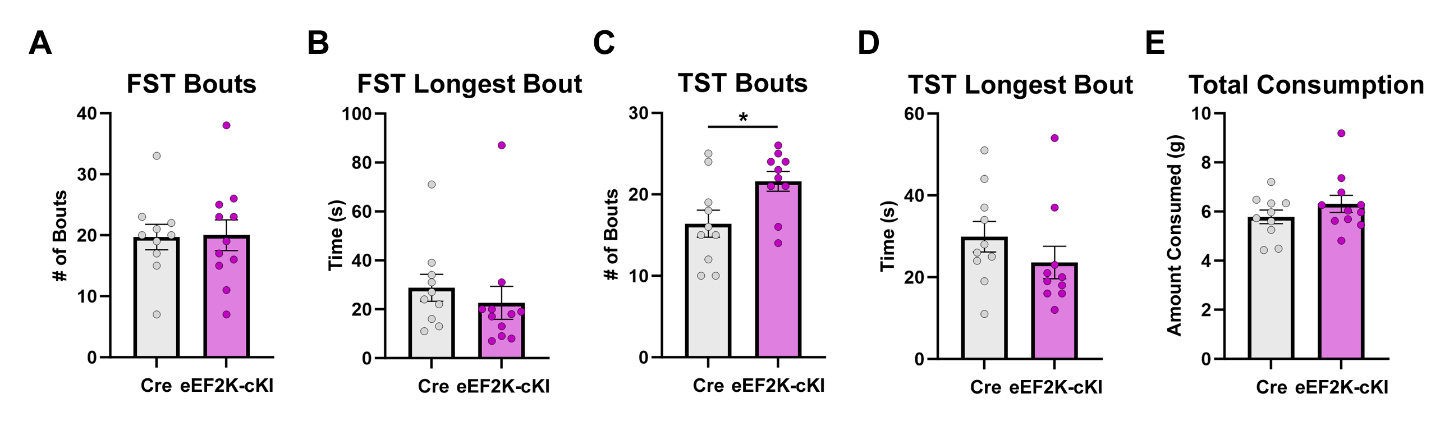


***Supplemental Figure 2: Control experiments for anhedonia and despair behaviors.*** (**A**) There was no change in the number of bouts of immobility between groups in the FST. Cre, n=10; eEF2K-cKI, n=11. p=0.9287. (**B**) There was no difference in the longest bout spent immobile between groups in the FST. Cre, n=10; eEF2K-cKI, n=11. p=0.4933. (**C**) eEF2K-cKI mice had a higher number of bouts of immobility in the TST. Cre, n=10; eEF2K-cKI, n=10. p=0.0210. (**D**) There was no difference in the longest bout spent immobile between groups in the TST. Cre, n=10; eEF2K-cKI, n=10. p=0.2644. (**E**) No change in the total amount consumed in the sucrose preference test between groups. Cre, n=10; eEF2K-cKI, n=11. p=0.2581. Error bars represent ± SEM. *p<0.05, Student’s T-test.


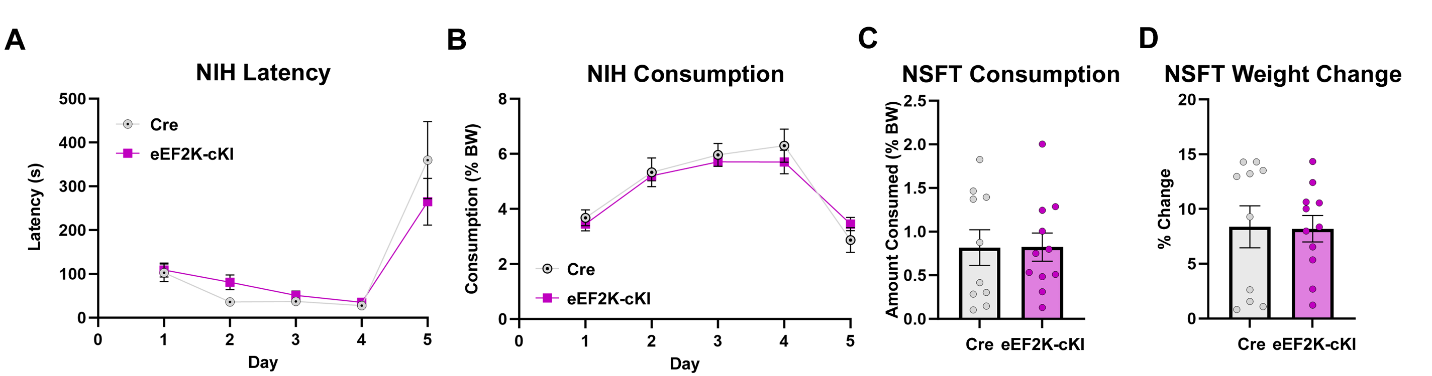


***Supplemental Figure 3: Control experiments for anxiety behaviors.*** (**A**) Latency during the training and testing days for NIH did not differ significantly between groups. (**B**) Amount consumed as % body weight over the training and testing days for NIH was not significantly different. Cre, n=10; eEF2K-cKI, n=11. (**C**) There was no difference between groups in the amount consumed in 5 min following NSFT. p=0.9852. (**D**) The percent weight change from before and after food deprivation for NSFT was not significantly different between groups. Cre, n=10; eEF2K-cKI, n=11. p=0.9373. Error bars represent ± SEM. Student’s T-test.


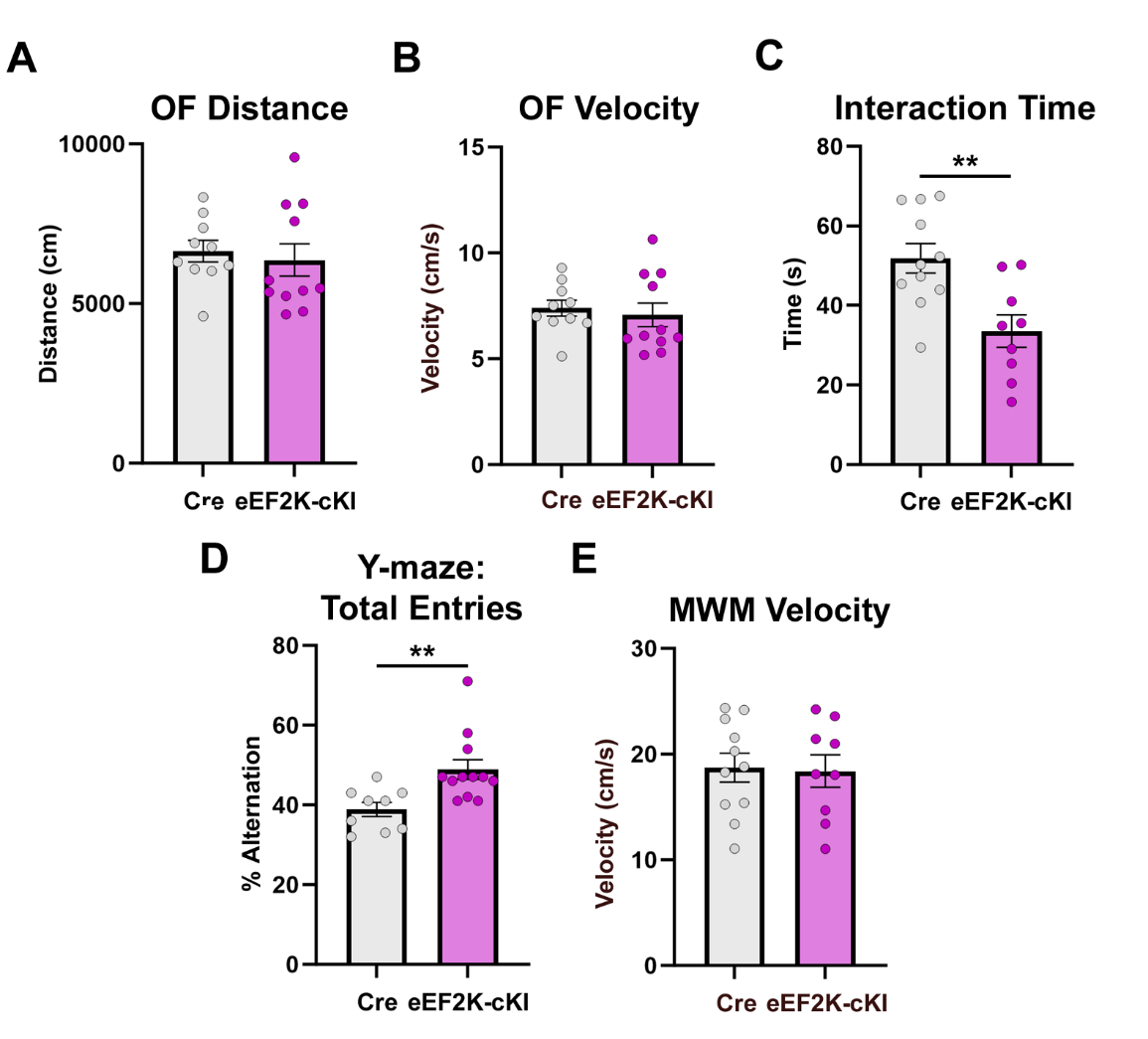


***Supplemental Figure 4: Control experiments for Learning and Memory Behaviors.*** (**A**) No change in the distance traveled during OF between groups. Cre, n=10; eEF2K-cKI, n=11. p=0.6588. (**B**) The average velocity during OF was not significantly different between groups. Cre, n=10; eEF2K-cKI, n=11. p=0.6531. (**C**) Decreased total interaction time for both objects in NOR in the eEF2K-cKI mice. Cre, n=11; eEF2K-cKI, n=9. p=0.0037. (**D**) Total arm entries in Y-maze task were increased in eEF2K-cKI mice. Cre, n=9; eEF2K-cKI, n=11. p=0.0059. (**E**) Average velocity during MWM probe trial was not significantly different between groups. Cre, n=11; eEF2K-cKI, n=9. p=0.8739. Error bars represent ± SEM. Student’s T-test.


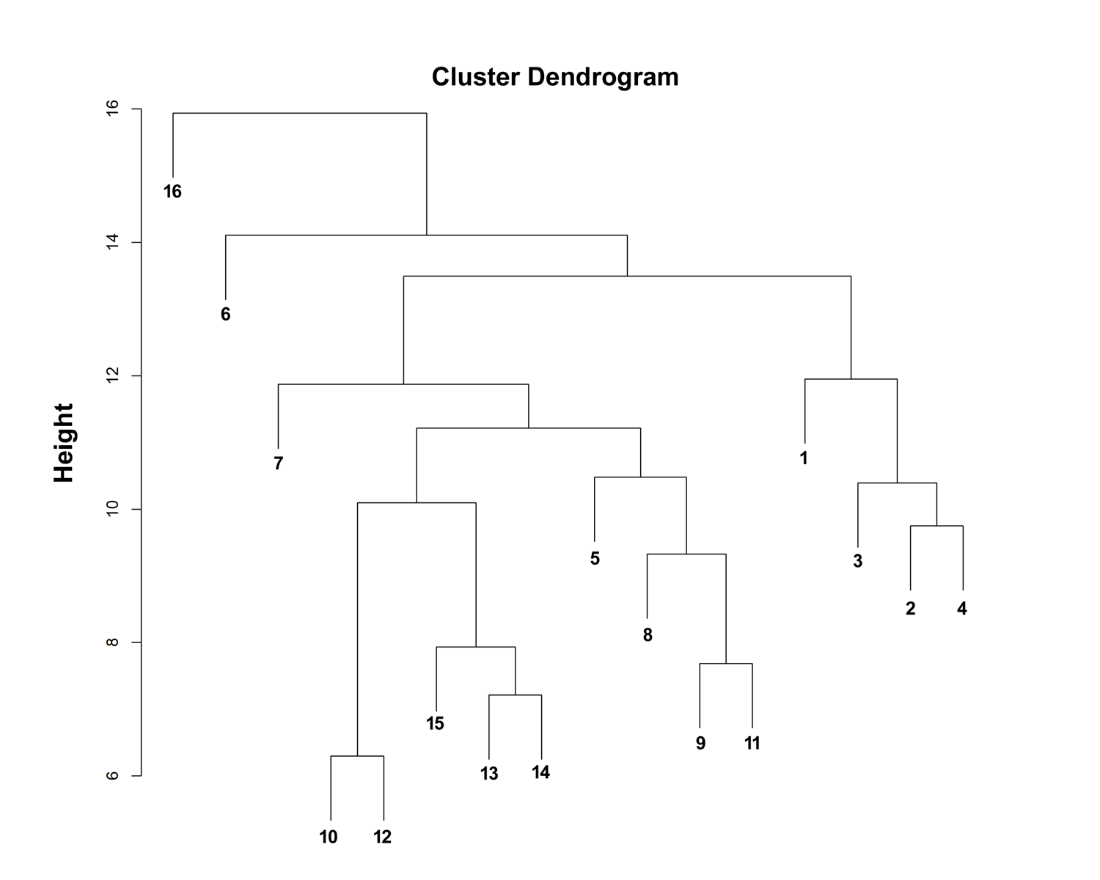


***Supplemental Figure 5: Hierarchical clustering and outlier identification in proteomics data.*** Cre, n=8; eEF2K-cKI, n=8. Samples 16 and 6 were removed as likely outliers.


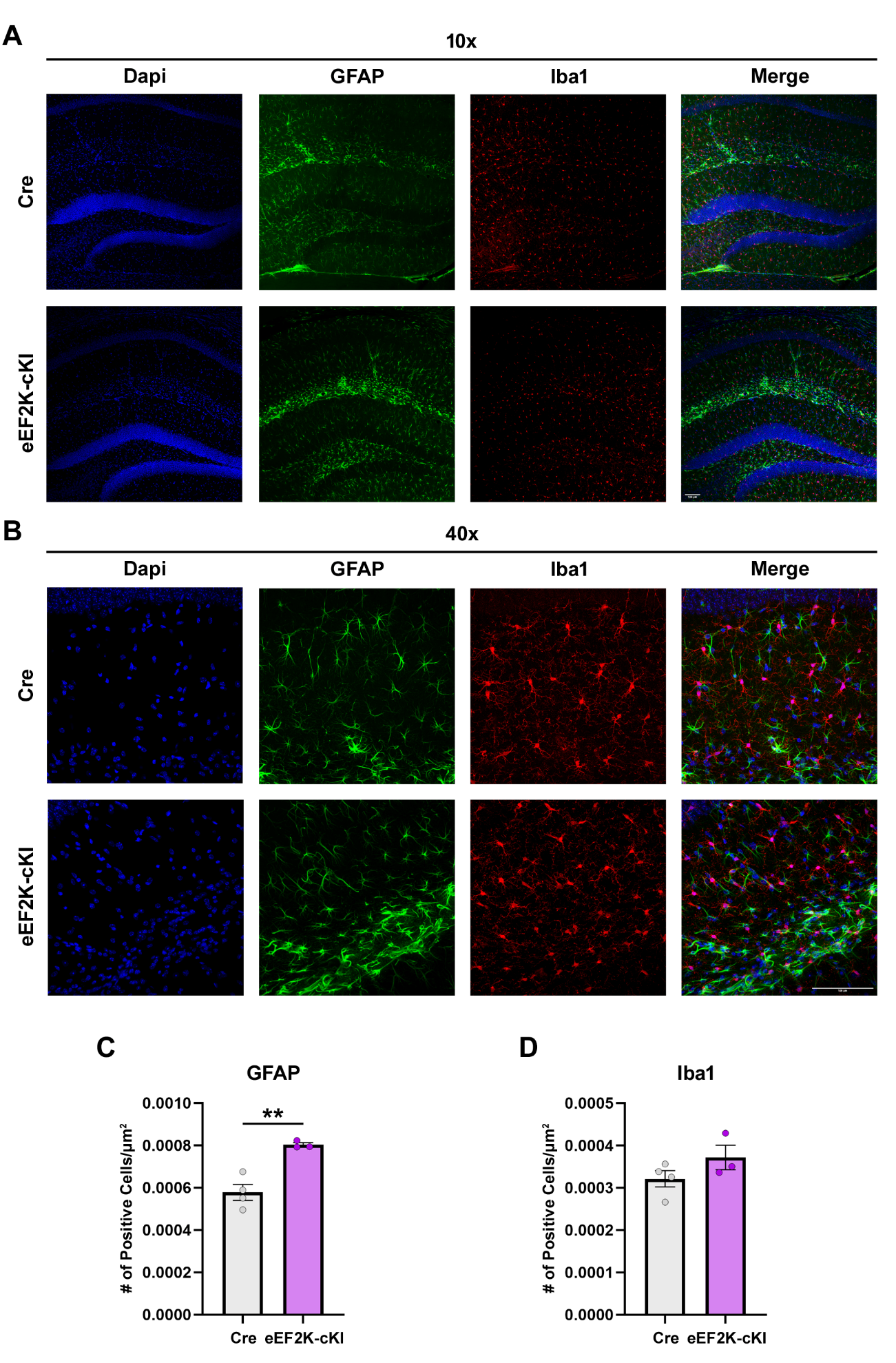


***Supplemental Figure 6: Iba1 and GFAP expression.*** (**A**) Representative immunofluorescence images of Dapi (blue), GFAP (green), Iba1 (red) in CA1 of the hippocampus. Scale bar = 100μm. Original magnification 10x. (**B**) Representative immunofluorescence images of Dapi (blue), GFAP (green), Iba1 (red) in CA1 of the hippocampus. Scale bar = 100μm. Original magnification 40x. (**C**) Increased number of GFAP-positive cells in eEF2K-cKI mice. p=0.0043. (**D**) No change in the number of Iba1-positive cells in eEF2K-cKI mice. p=0.1920. n=2 mice per group with 1-2 images per mouse. Error bars represent ± SEM. **p<0.01; Student’s T-test.
